# Supplementary material for: TGFβ promotes widespread enhancer chromatin opening and operates on genomic regulatory domains
Source: Nat Commun. 2020 Dec 3;11:6196. doi: 10.1038/s41467-020-19877-5 (PMC7713251; doi:10.1038/s41467-020-19877-5)

TGFβ Promotes Widespread Enhancer Chromatin Opening and Operates on Genomic Regulatory Domains

Jose A. Guerrero-Martínez, María Ceballos-Chávez, Florian Koehler, Sandra Peiró, and Jose C. Reyes

Supplementary Figure 1. Analysis of ATAC-seq and DNaseI-seq experiments

Supplementary Figure 2. Characterization of TGFβ-mediated increase of chromatin accessibility by ATAC-seq and ATAC-see

Supplementary Figure 3. Analysis of transcription factors occupancy to enhancers with different changes in chromatin accessibility upon TGFβ treatment.

Supplementary Figure 4. Classification of genes in categories depending on differential expression after TGFβ treatment

Supplementary Figure 5. Classification of enhancers depending on histone posttranslational modifications

Supplementary Figure 6. Correlation between H3K27Ac and eRNA Levels

Supplementary Figure 7. Analysis of SMAD2/3 ChIP-seq data

Supplementary Figure 8. Most TGFβ-dependent genes are in the enhancer's neighborhood

Supplementary Figure 9. Characterization of changes in gene expression around a regulated enhancer

Supplementary Figure 10. TGFβ-regulated genes often appear in clusters of co-regulated genes.

Supplementary Figure 11. Changes of H3K4me3 around TGFβ–regulated genes

Supplementary Figure 12. Characterization of TRD and non-TRD genes

Supplementary Figure 13. Verification by ChIP-PCR of sgRNA targeting and dCas9-KRAB repression activity

Supplementary Figure 14. Controls for CRISPR-mediated enhancer inactivation.

Supplementary Figure 15. TGFβ-dependent regulation is affected by TAD borders.

Supplementary Figure 16. CTCF distribution at TAD and TRD borders

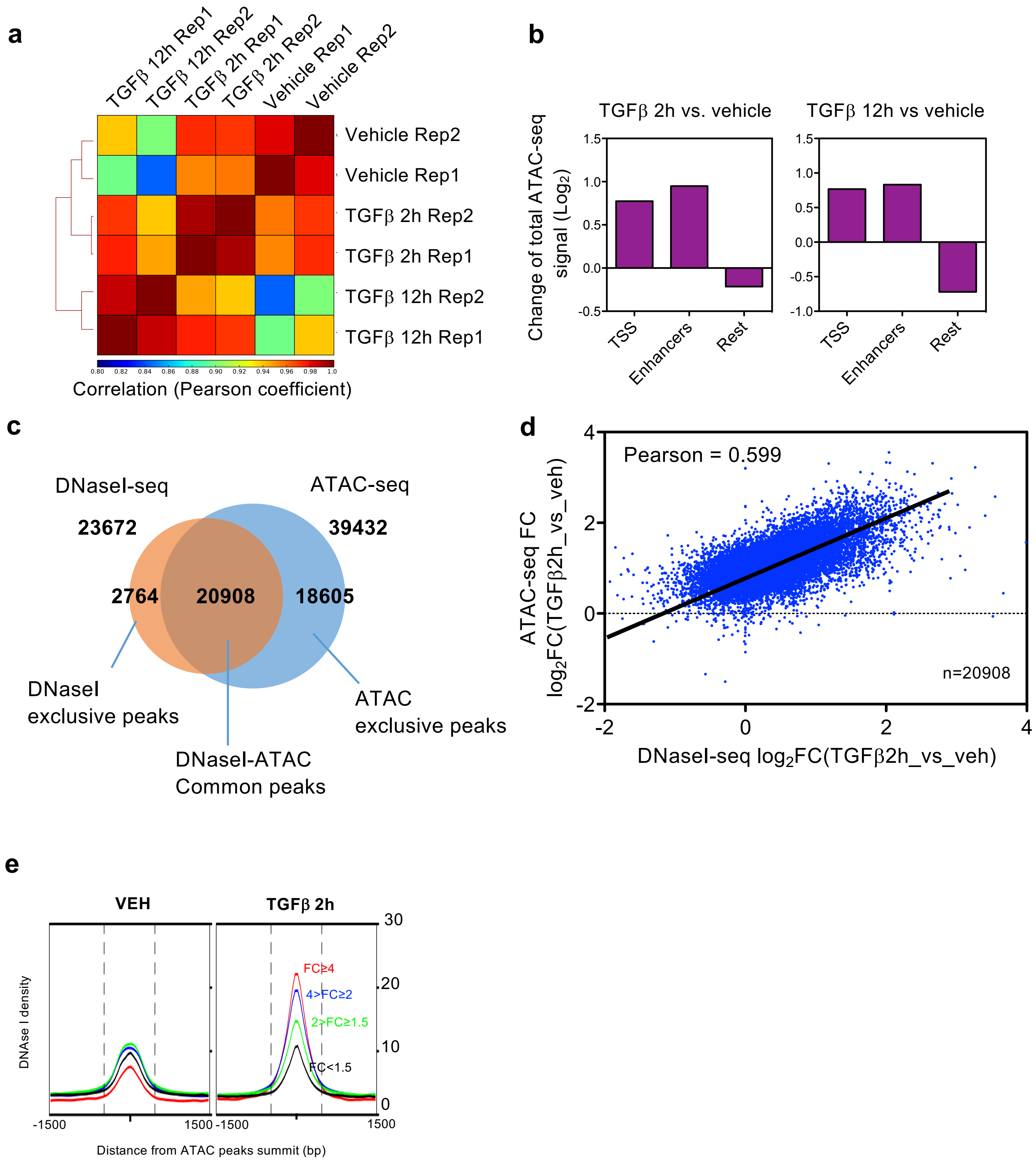

Supplementary Figure 1

Supplementary Figure 1. Analysis of ATAC-seq and DNaseI-seq experiments

a Correlation between replicates of ATAC-seq experiments. **b** Change of total genomic ATAC-seq signal in promoters (TSS), enhancers, and the rest of the genome; 2h TGFβ treatment versus vehicle (left) and 12h TGFβ treatment versus vehicle (right) are shown. **c** Overlapping between DNaseI-seq peaks and ATAC-seq peaks. **d** Correlation between changes of ATAC-seq and DNaseI-seq signals upon TGFβ addition (log_2FC(TGFβ2h_vs_veh)), of the 20908 common regions of open chromatin found using both techniques. **e** DNaseI-seq signal density plots of vehicle and 2h TGFβ at non-TSS ATAC-seq peaks. The four curves correspond to the ATAC-peaks categories defined in Fig. 2a.

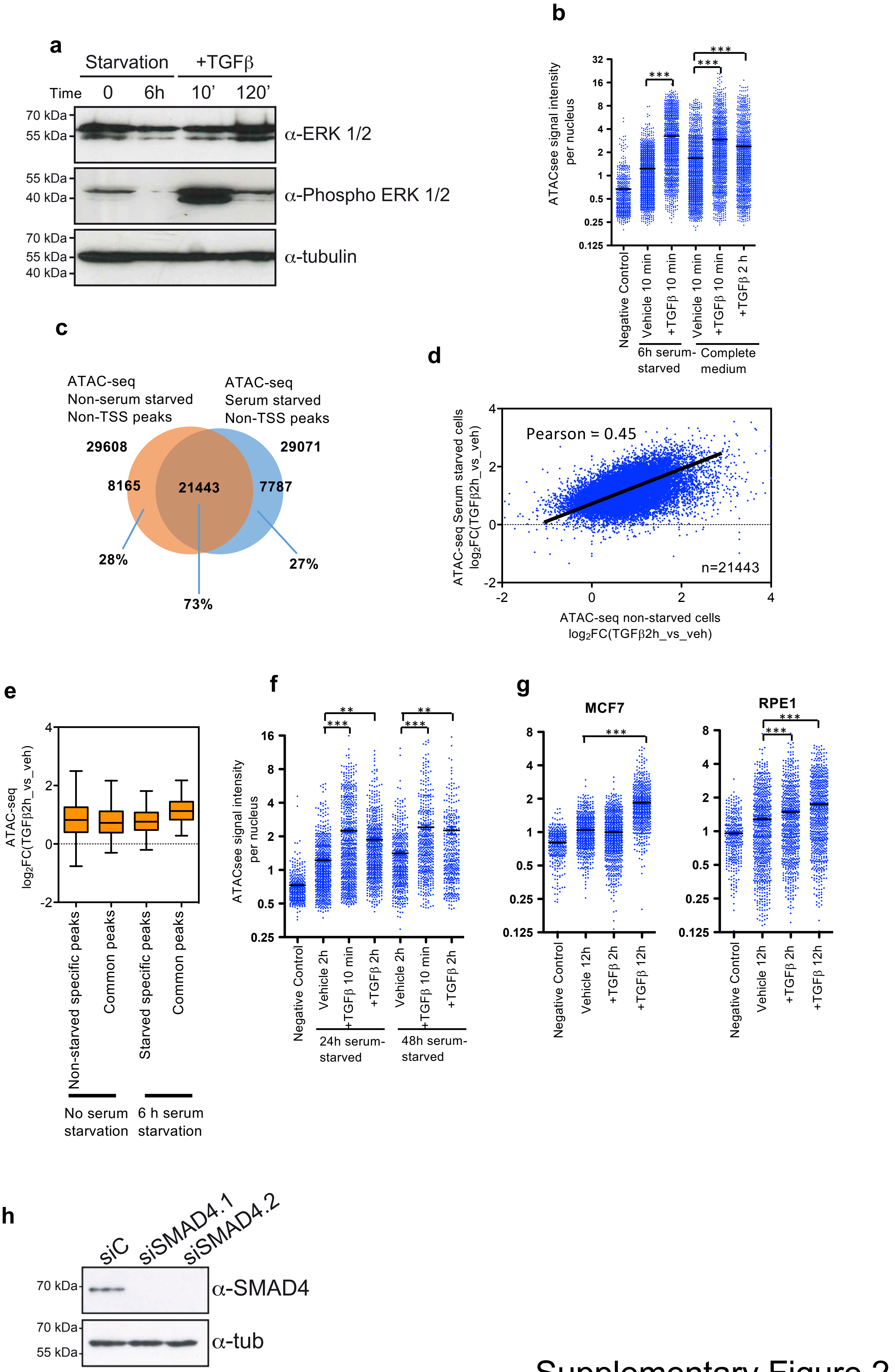

Supplementary Figure 2

Supplementary Figure 2. Characterization of TGFβ-mediated increase of chromatin accessibility by ATAC-seq and ATAC-see.

a Western blotting showing activation of the Erk pathway by TGFβ addition at the indicated time points, after 6h of serum and insulin starvation, in NMuMG cells. Representative images out of three independent experiments are shown. **b** Quantification of ATAC-see signal intensity of nuclei at the indicated times after TGFβ or vehicle addition. Serum starved cells were deprived from serum (and insulin) during 6h before TGFβ treatment, while non-starved cells were maintained in the complete growth medium before treatment. **c-e** Comparison of TGFβ-dependent accessibility change, determined by ATAC-seq, in 6h-serum starved versus non-starved cells. Starvation conditions as described in **b**. **c** Overlapping between non-TSS ATAC-seq peaks from 6h-serum starved or non-starved cells treated with TGFβ. **d** Correlation between changes of ATAC-seq signal upon TGFβ addition (log_2FC(TGFβ2h_vs_veh)) of the 21443 common regions of non-TSS open chromatin between 6h-serum starved or non-starved cells. A strong positive correlation between the two set of data was observed. **e** Boxplot showing changes of ATAC-seq signal upon TGFβ addition (log_2FC(TGFβ2h_vs_veh)) of the indicated subset of peaks shown in **c** in 6h-serum starved or non-starved cells. The vast majority (>80%) of the changes were > 0 in all the cases, consistent with the pervasive chromatin opening provoked by TGFβ. The horizontal black line of the boxplot represents the median value, the box spans the 25th to 75th percentiles, and whiskers indicate 5th and 95th percentiles. Non-starved specific, n=8165; Non-starved common, n = 21443; Starved specific, n=7787; Starved common, n= 21443. **f, g** Quantification of ATAC-see signal intensity of nuclei at the indicated times after TGFβ or vehicle addition. **f** NMuMG cells were serum (and insulin) starved during 24h or 48h before TGFβ addition. **g** MCF7 or RPE1 cells were serum starved during 24h before TGFβ addition. **h** Western blot showing knockdown of Smad4 using two different siRNAs. NMuMG cells were transfected with either siControl or one of the two different siRNAs that target Smad4. 48h after transfection, protein extracts were analyzed by Western blotting using the antibodies indicated on the left. **b, e-g** Statistical significances of the difference between the indicated and the vehicle (control) distributions were determined by using the two-tailed Mann-Whitney non-parametric test. *$p \leq 0.05$; **$p \leq 0.01$; ***$p \leq 0.001$. Number of elements in each scatter dot blot or boxplot and exact p-values are provided in Supplementary Data 5. The horizontal black line of the scatter blot represents the mean value.

a

	Family of footprints	FC≥4 (n= 1113)	2≤FC<4 (n = 14222)	1.5≤FC<2 (n= 8926)	FC<1.5 (n=4810)
2h TGFβ	AP-1	2.27×10^{-106}	$\leq 10^{-300}$	1.76×10^{-120}	2.42×10^{-16}
	SMAD3/4	1.55×10^{-09}	0.009	0.18	0.34
	CTCF/CTCFL	0.05	1.87×10^{-11}	1.20×10^{-163}	1.02×10^{-176}
10 min TGFβ	**Family of footprints**	**FC≥4 (n= 527)**	**2≤FC<4 (n = 9368)**	**1.5≤FC<2 (n= 5341)**	**FC<1.5 (n=1875)**
	AP-1	0.17	3.79×10^{-63}	1.70×10^{-23}	5.03×10^{-8}
	SMAD3/4	1	1	0.25	1
	CTCF/CTCFL	0.0001	3.36×10^{-66}	8.92×10^{-39}	1.88×10^{-11}
	ETS	0.02	0.003	6.44×10^{-5}	1

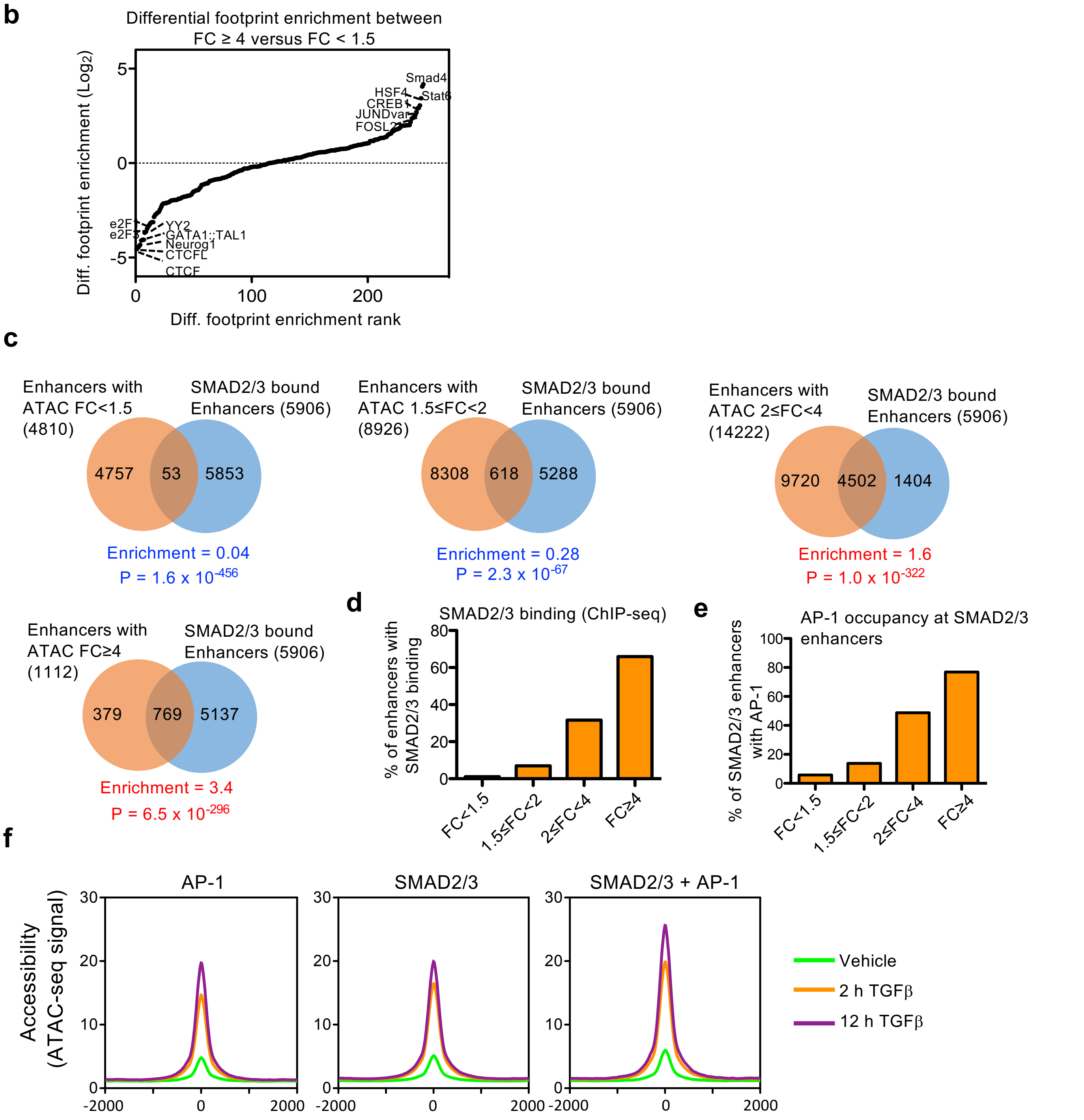

Supplementary Figure 3

Supplementary Figure 3. Analysis of transcription factors occupancy to enhancers with different changes in chromatin accessibility upon TGFβ treatment.

a P-value (one-sided Fisher's exact test) of the enrichment of the indicated family of footprints in the different categories of enhancers, classified depending on change of accessibility at 2h after TGFβ, versus random regions. **b** Differential footprint enrichment between enhancers with change of accessibility FC ≥ 4 and FC < 1.5 (12h after TGFβ vs vehicle). **c** Overlap between enhancers that present SMAD2/3 binding (by ChIP-seq, 1.5 h after TGFβ from GEO: GSE121254) and enhancers classified according to their increase of accessibility 2h after TGFβ. Positive (red) and negative (blue) enrichment with the corresponding probability (p) using hypergeometric distributions are shown. **d** Percentage of enhancers of each category (according to increase of accessibility at 2h after TGFβ) that contain a SMAD2/3 ChIP-seq peak. **e** Percentage of SMAD2/3 containing enhancers of each category (according to increase of accessibility 2h after TGFβ) that contain AP-1 footprints. **f** ATAC signal density around the ATAC peak summit of enhancers with an AP-1 footprint (left), SMAD2/3 binding as assayed by ChIP-seq (middle), or both together (right).

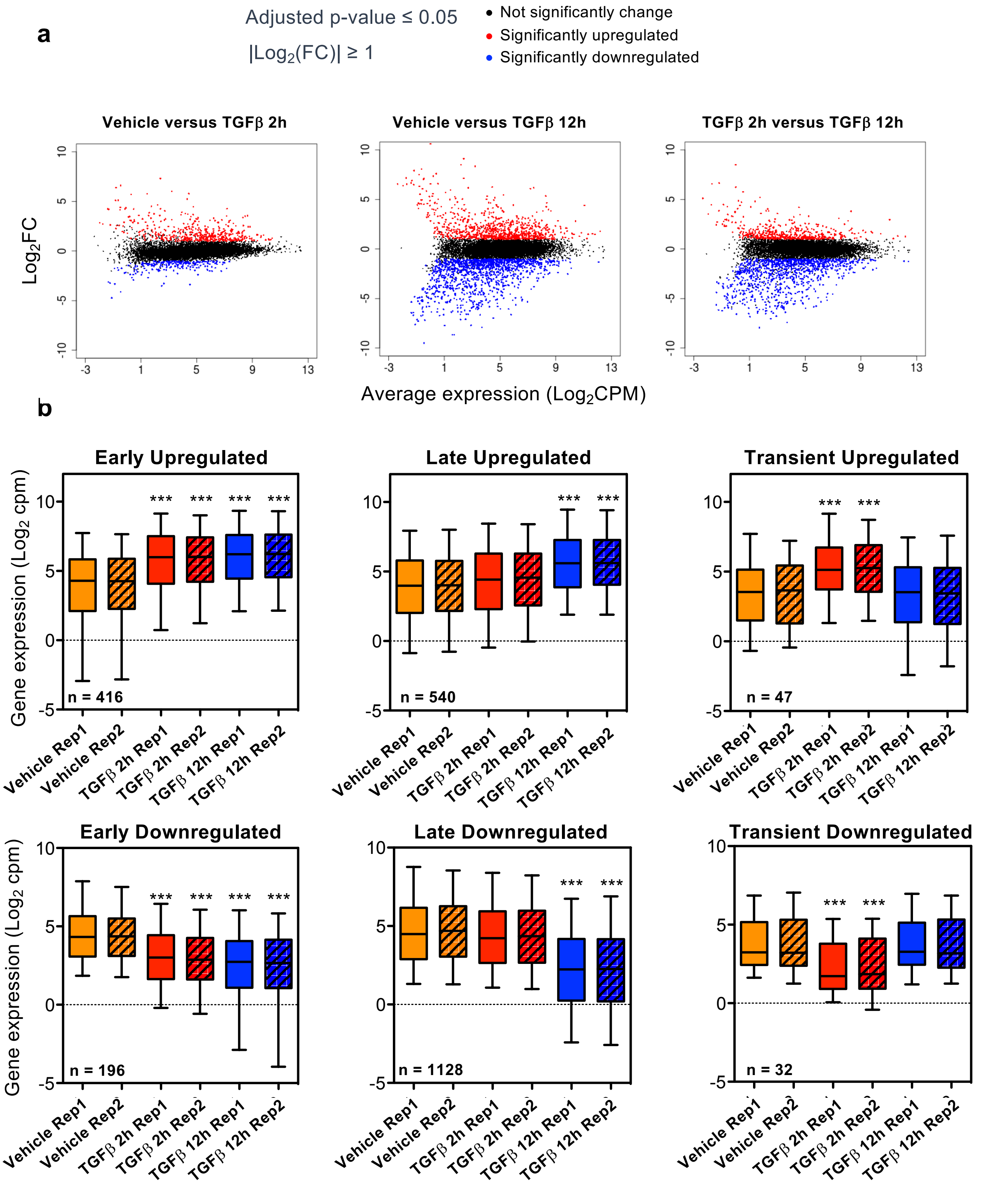

Supplementary Figure 4

Supplementary Figure 4. Classification of genes in categories depending on their differential expression after TGFβ treatment

RNA-seq experiments were performed following the flow diagram shown in Figure 1a. **a** MA plots showing significant differentially expressed genes with adjusted p-value < 0.05 and $|\log_2 FC| > 1$, comparing the indicated conditions. **b** Boxplot of total expression (as CPM) of genes from every category. Data of both independent replicates are shown. Number of differentially regulated genes in each category (n) is provided. Statistical significances of the difference between the indicated and the vehicle distributions were determined by using the two-tailed Mann-Whitney non-parametric test. ***$p \leq 0.001$. Exact p-values are provided in Supplementary Data 5. The horizontal black line of the boxplot represents the median value, the box spans the 25th to 75th percentiles, and whiskers indicate 5th and 95th percentiles.

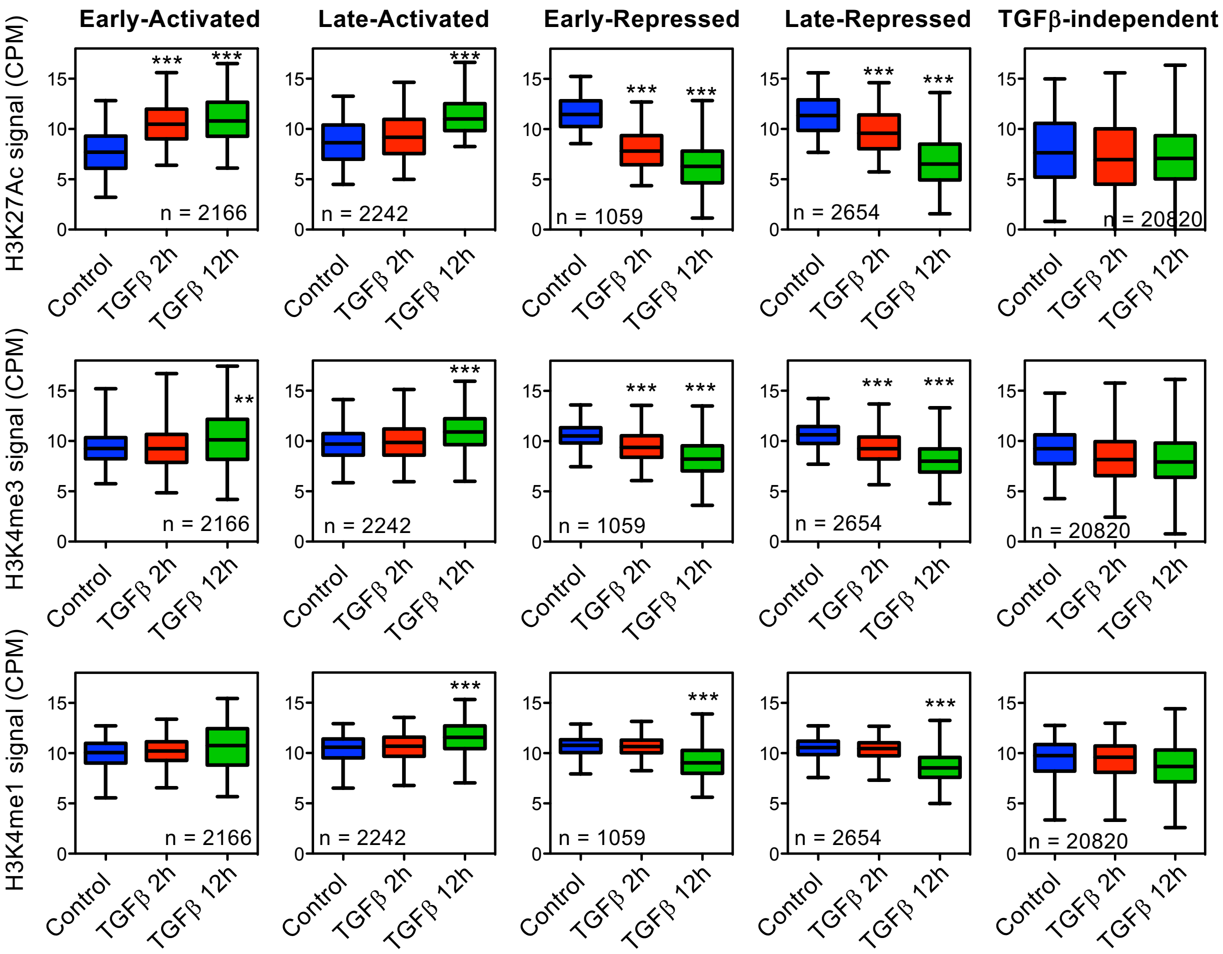

Supplementary Figure 5. Classification of enhancers depending on histone posttranslational modifications

H3K27Ac, H3K4me3 and H3K4me ChIP-seq signals (as CPM) of the different categories of enhancers shown in the heatmap of Figure 3a were quantified and represented in boxplots. CPM are the average of two independent replicates. The number of genes (n) in each category is provided. Statistical significances of the difference between the indicated and the vehicle (control) distributions were determined by using the two-tailed Mann-Whitney non-parametric test. *p ≤ 0.05; **p ≤ 0.01; ***p ≤ 0.001. Exact p-values are provided in Supplementary Data 5. The horizontal black line of the boxplot represents the median value, the box spans the 25th to 75th percentiles, and whiskers indicate 5th and 95th percentiles.

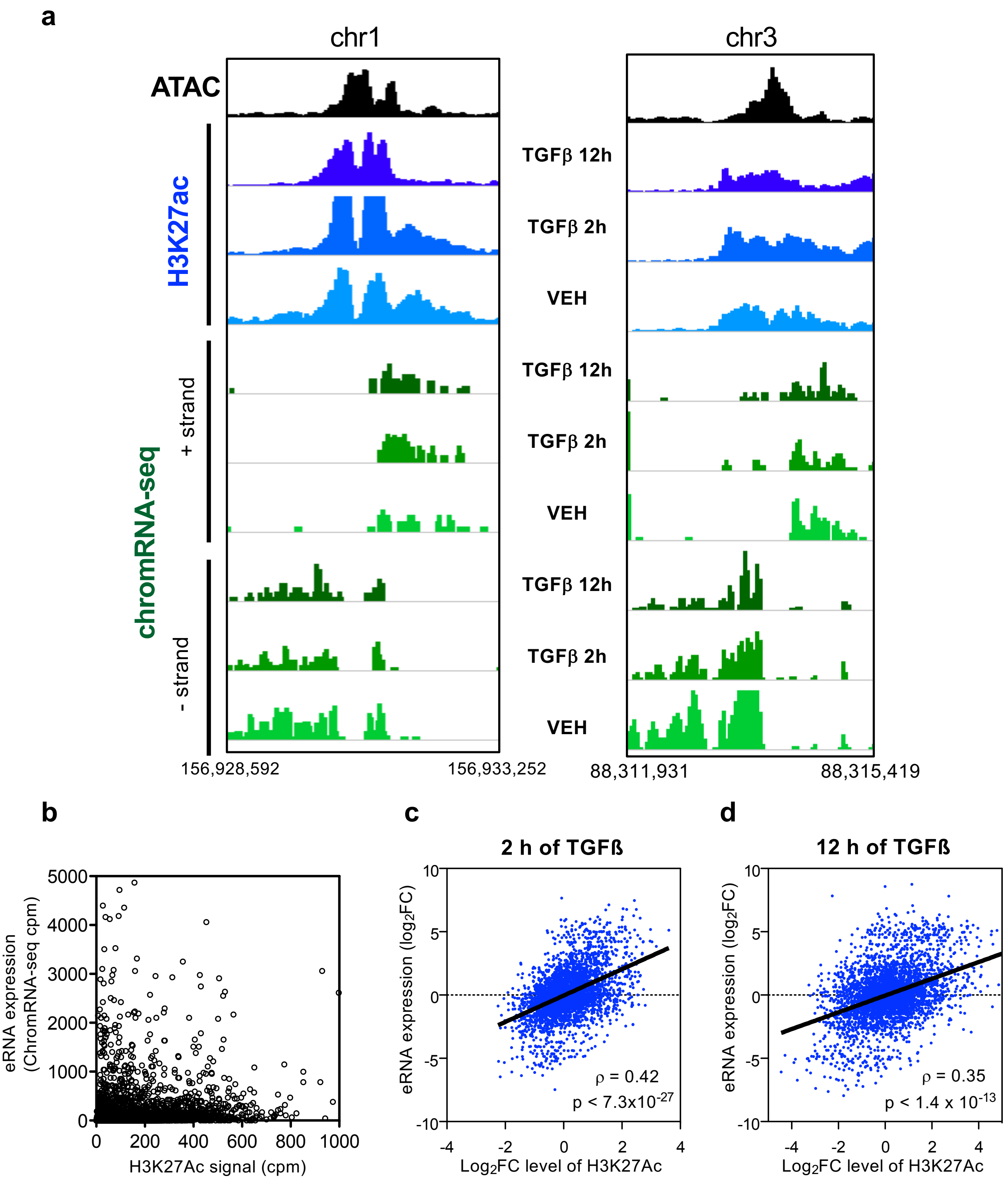

Supplementary Figure 6. Correlation between H3K27Ac and eRNA Levels
a Example of bidirectional eRNAs expressed from enhancers. Screenshot of chromRNA-seq tracks at two different genomic regions. **b** Correlation plot between total level of eRNA and level of H3K27Ac (ChIP-seq signal as CPM) prior to TGFβ addition. For eRNA, ChromRNA-seq signal (as CPM) associated to a 400 bp window surrounding the ATAC-seq peak (summit ± 200 bp) was used. To avoid quantification of mRNA or pre-mRNA in the determination of eRNA, only intergenic enhancers were considered. **c, d** Correlation plot between total change of eRNA levels and change of H3K27Ac levels 2h (**c**) or 12h (**d**) after TGFβ addition. Spearman correlation coefficients and p-values are provided.

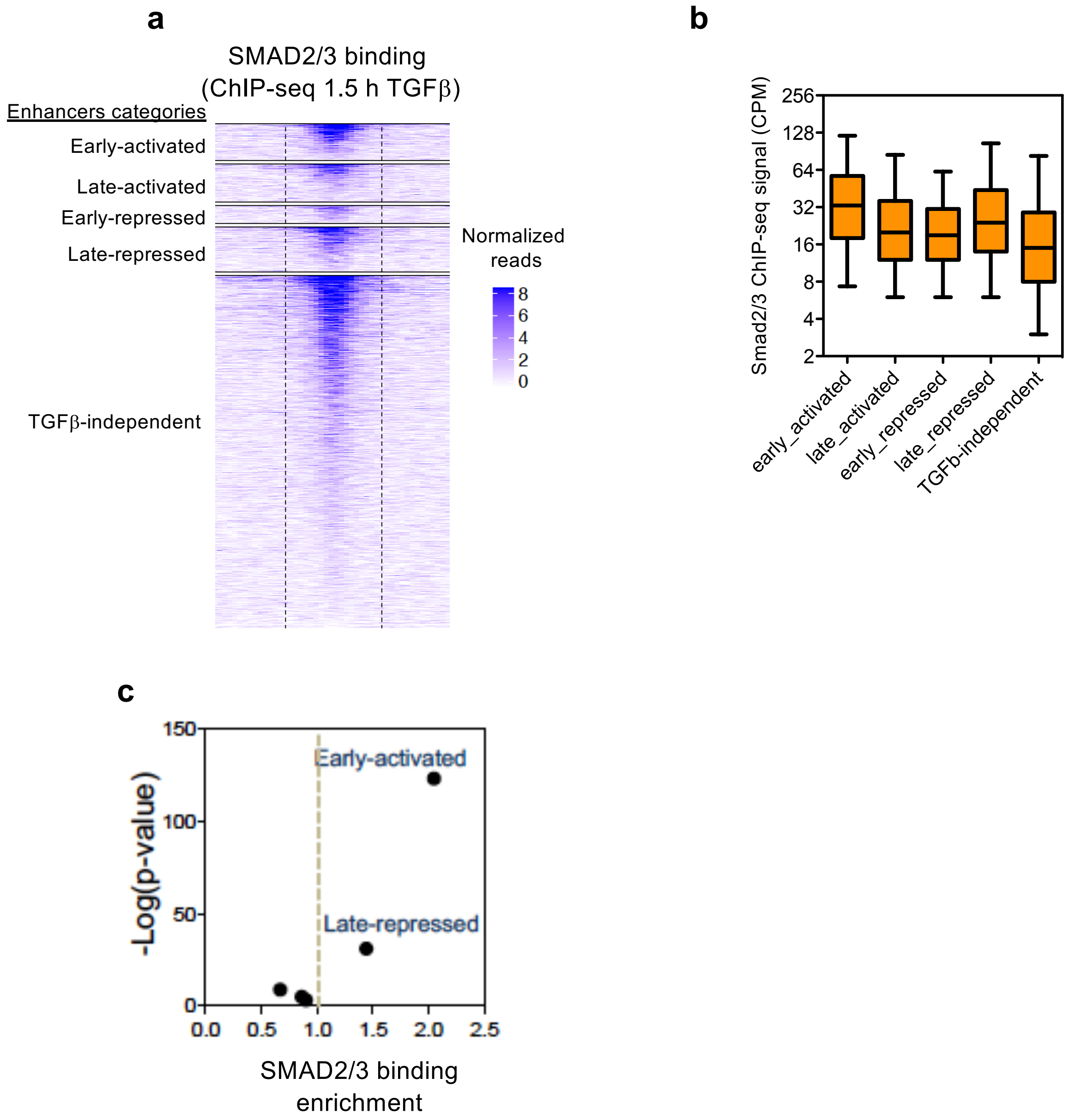

Supplementary Figure 7. Analysis of SMAD2/3 ChIP-seq data.

a Heatmap showing SMAD2/3 ChIP-seq signal of NMuMG cell treated with TGFβ during 1.5h (GEO: GSE121254). Enhancers were classified as in Figure 3a. **b** Boxplot showing quantification of SMAD2/3 ChIP-seq signal in the indicated enhancer categories. Early activated, n=2166; Late activated, n=2242; Early repressed, n=1059; Late repressed, n=2654; TGFβ-independent, n=20820, where number of data correspond to the number of enhancers in each category. The horizontal black line of the boxplot represents the median value, the box spans the 25th to 75th percentiles, and whiskers indicate 5th and 95th percentiles. **c** Enrichment of SMAD2/3 ChIP-seq peaks in the different enhancer categories with respect to the rest of categories. On the y-axes, $-\log_{10}$ (p-value) of enrichment (Hypergeometric distribution).

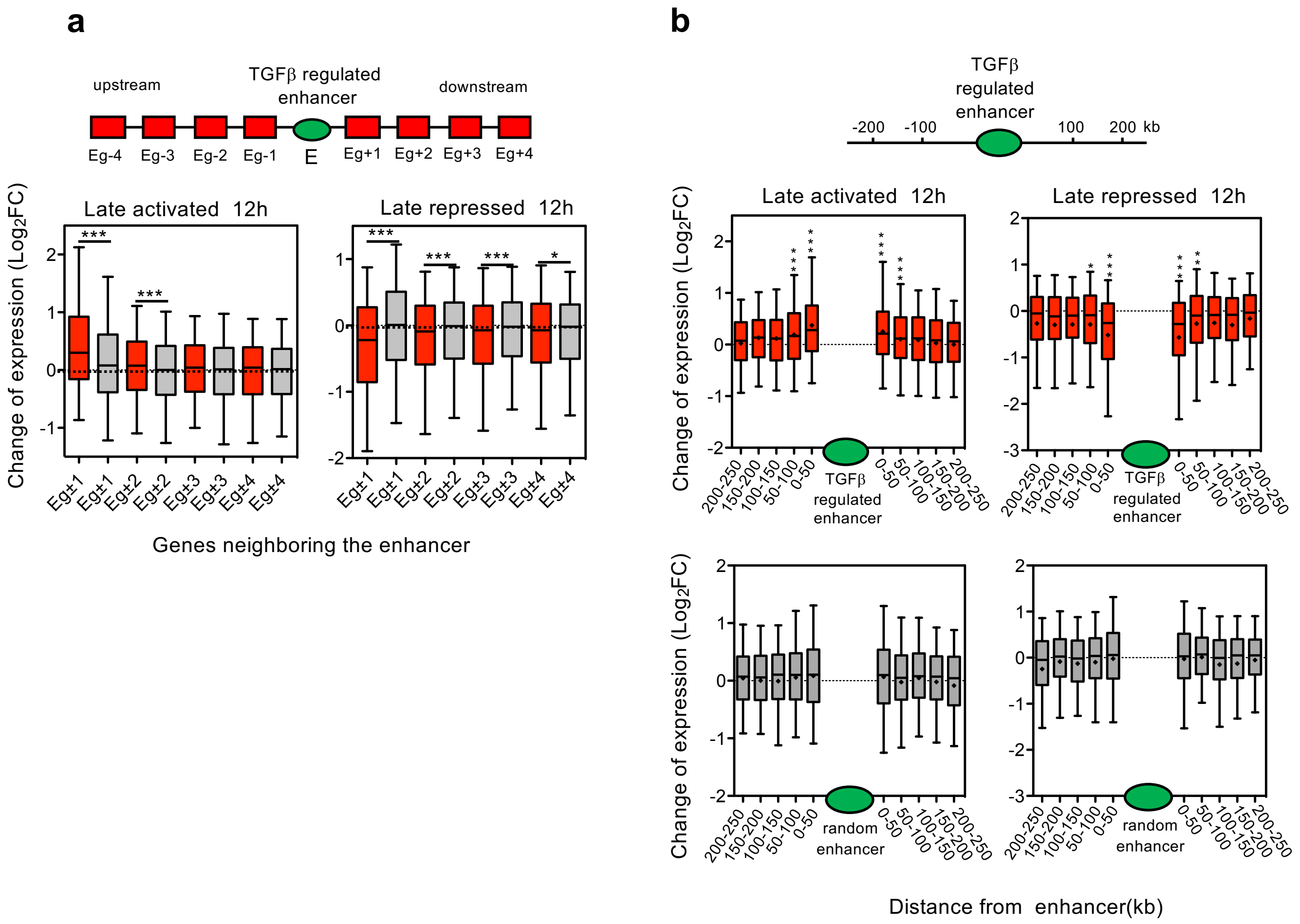

Supplementary Figure 8. Most TGFβ-dependent genes are in the enhancer's neighborhood

a Boxplot showing changes of mRNA levels (RNA-seq signal, TGFβ 12 h versus vehicle) of genes located upstream and downstream of a late-activated (left) or late-repressed (right) enhancer, as in the scheme (red), or upstream and downstream of a randomly selected enhancer (grey). For randomization see Methods. Eg±n with n=1, 2, 3 and 4 mean genes that occupy the first, second, third, or fourth position, respectively, in the chromosomal order, upstream (–) or downstream (+) of the enhancer. **b** Boxplot showing change of mRNA level (RNA-seq signal after TGFβ versus vehicle for 12 h) of genes located at the indicated distance (kb) upstream and downstream of late-activated (top left) or late-repressed (top right) enhancers. Inclusion of genes in the interval was determined by the position of its TSS. Lower panels represent the corresponding data for randomized enhancers (see Methods). **a, b** Statistical significance of the difference between real and random distributions were determined by using the two-tailed Mann-Whitney non-parametric test. *$p \leq 0.05$; **$p \leq 0.01$; ***$p \leq 0.001$. Number of data in each boxplot and exact p-values are provided in Supplementary Data 5. The horizontal black line of the boxplot represents the median value, a dot represents the mean, the box spans the 25th to 75th percentiles, and whiskers indicate 5th and 95th percentiles.

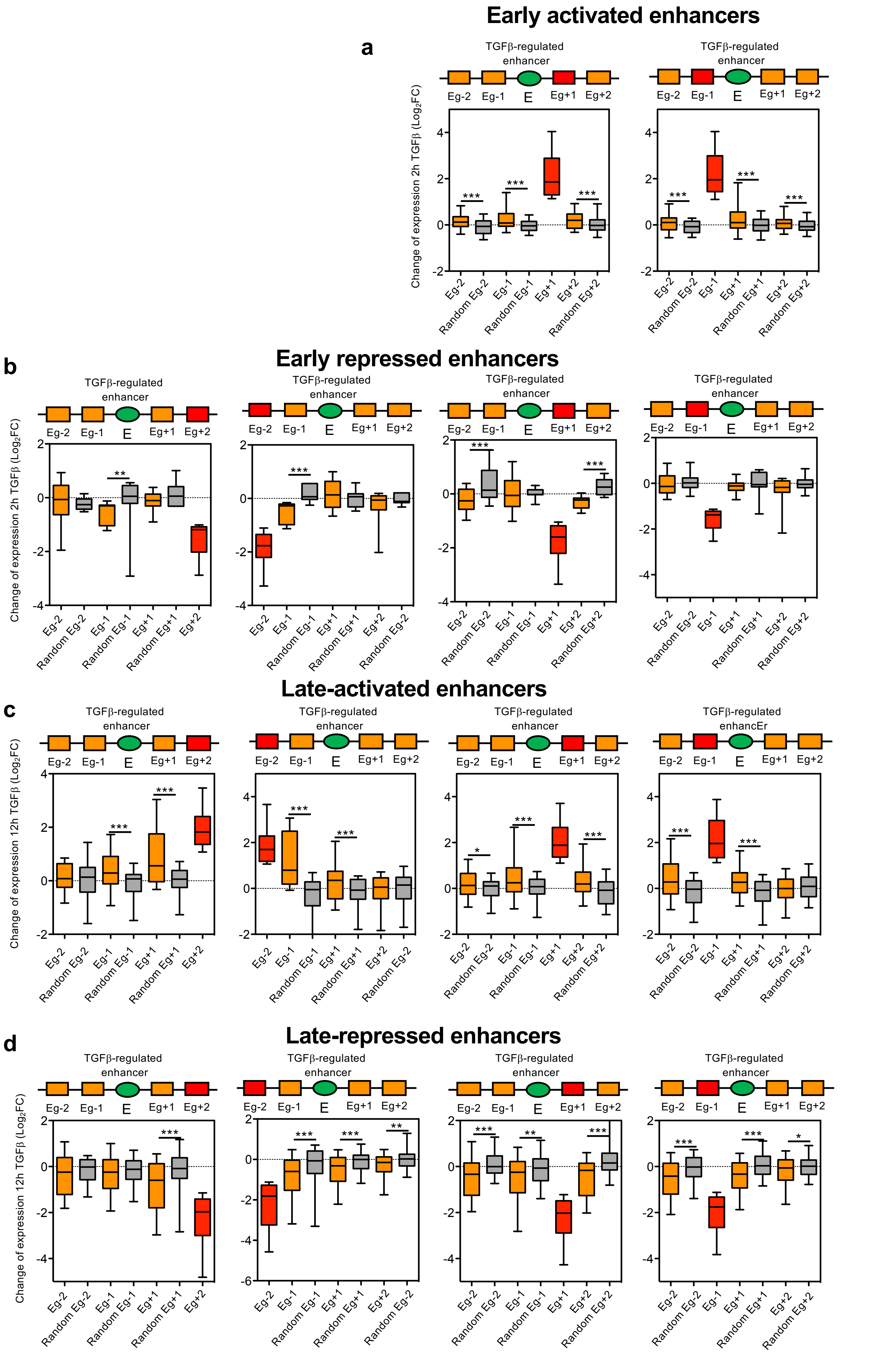

Supplementary Figure 9

Supplementary Figure 9. Characterization of changes in gene expression around a regulated enhancer

Changes in mRNA levels (RNA-seq signal) of genes around an early-activated (**a**), early-repressed (**b**), late-activated (**c**), or late-repressed (**d**) enhancer. Only enhancers for which the gene marked in red was robustly regulated ($|\log_2 FC| > 1$; adjusted $p < 0.05$) were considered. Grey boxes contain identical data considering randomized enhancers. Statistical significance between real and random distributions were determined with the two-tailed Mann-Whitney non-parametric test. *$p \leq 0.05$; **$p \leq 0.01$; ***$p \leq 0.001$. Number of data (n) in each boxplot and exact p-values are provided in Supplementary Data 5. The horizontal black line of the boxplot represents the median value, the box spans the 25th to 75th percentiles, and whiskers indicate 5th and 95th percentiles.

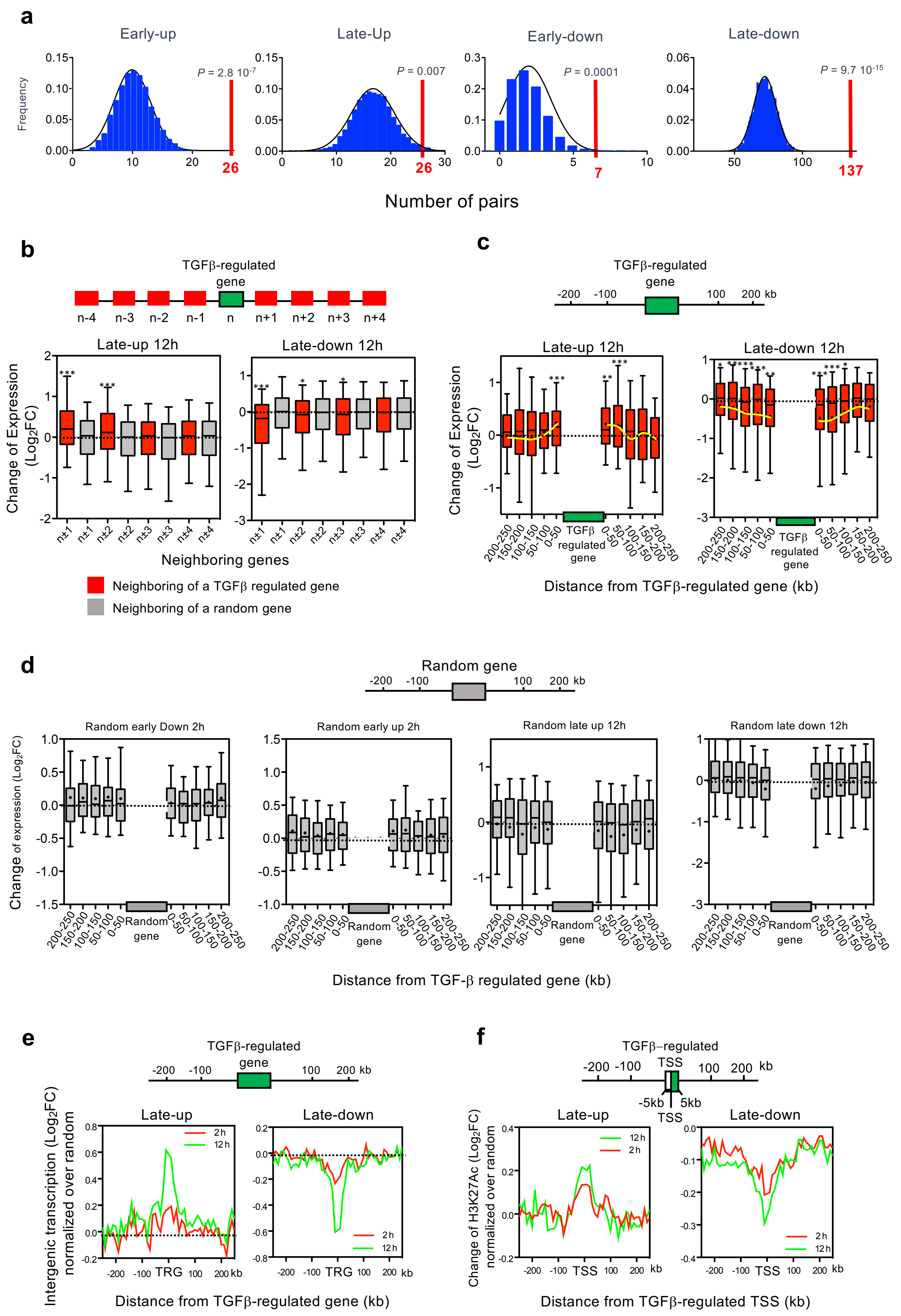

Supplementary Figure 10

Supplementary Figure 10. TGFβ-regulated genes often appear in clusters of co-regulated genes. a, Number of pairs of genes robustly co-regulated by TGFβ (both genes regulated by TGFβ in the same way, with $|log_2FC| > 1$; adjusted $p < 0.05$). To determine statistical significance, the numbers of real pairs were compared with 5000 randomizations of the gene order. Histograms show the distribution of the number of pairs obtained from the randomized orders. Red bars correspond to the real number of pairs of co-regulated genes. Probabilities (p) of the real number considering Normal distribution are provided. **b** Boxplot showing the change of mRNA levels (RNA-seq) of the four genes located upstream (n-1 to n-4) or downstream (n+1 to n+4) of a robustly TGFβ-regulated gene (log_2FC of the regulated gene ≥ 1; adjusted p ≤ 0.05) at position n. The central gene (n) is late-upregulated (left) or late-repressed (right). Grey boxes correspond to changes of mRNA levels of genes around a random gene. **c** Boxplot showing changes of mRNA levels (RNA-seq) of genes located at the indicated distance (kb) upstream and downstream of a TGFβ-regulated gene. The central gene is late-upregulated (left) or late-repressed (right). Statistical significance between real and random distributions were determined by using the two-tailed Mann-Whitney test, *p ≤ 0.05; **p ≤ 0.01; ***p ≤ 0.001. **d** Boxplot showing changes of mRNA levels (RNA-seq) of genes located at the indicated distance (kb) upstream and downstream of a random gene, for the indicated categories of genes. **b-d** Number of data (n) in each boxplot and exact p-values are provided in Supplementary Data 5. The horizontal black line of the boxplot represents the median value, and the dot represents the mean. The box spans the 25th to 75th percentiles, and whiskers indicate 5th and 95th percentiles. **e** Changes of intergenic transcription in the neighborhood (± 250 kb binned in 10 kb bins) of a TGFβ-regulated gene (TRG) normalized to random. ChromRNA-seq data were used. To avoid termination read-thought and promoter-divergent transcription, regions of 2 kb upstream and downstream of the TSS and the transcription termination site, respectively, were not considered. **f** Change of H3K27ac levels (ChIP-seq signal) in the neighborhood (± 250 kb binned in 10 kb bins) of a TGFβ-regulated TSS normalized to random. To avoid consideration of histone modifications of the TGFβ-regulated TSS, regions of 5 kb upstream and downstream of the TSS were not considered. **b, c, e, f**, Early-upregulated and early-repressed categories are shown in Figure 5c, d, e, f, respectively. **a-f** For randomizations see Methods.

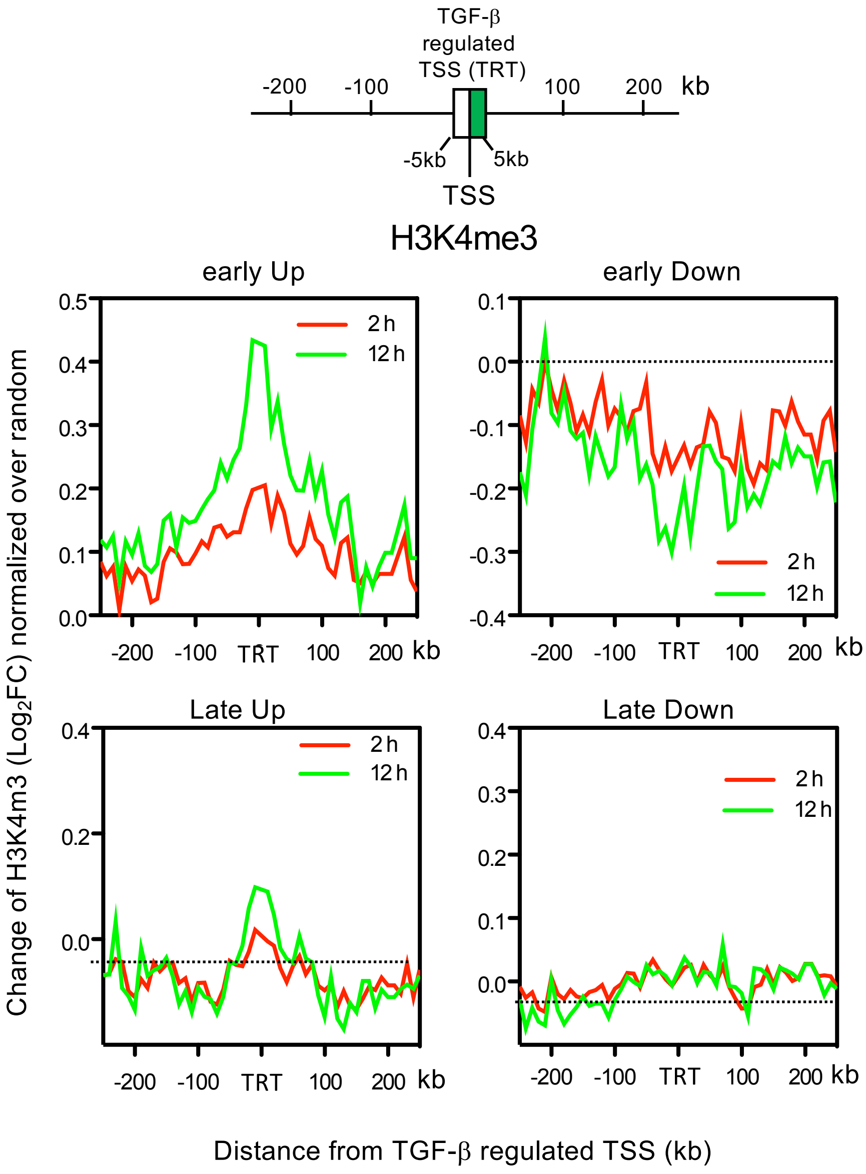

Supplementary Figure 11. Changes of H3K4me3 around TGFβ–regulated genes
Change of H3K4me3 levels (ChIP-seq signal) in the neighborhood (± 250 kb binned in 10 kb bins) of a TGFβ-regulated TSS normalized to random (see Methods). To avoid histone modifications of the TGFβ-regulated TSS, regions of 5 kb upstream and downstream of the TSS were not considered.

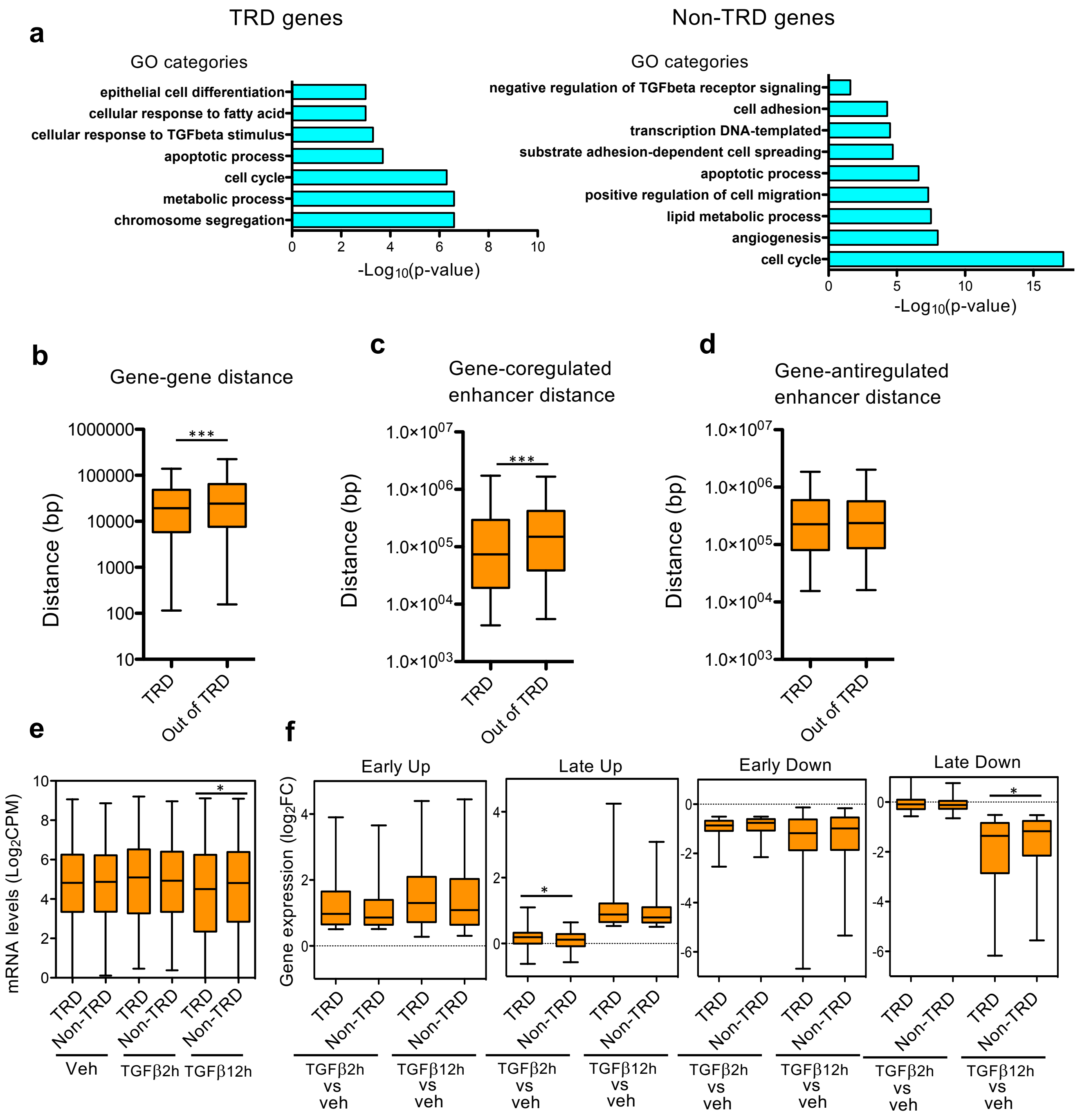

Supplementary Figure 12

Supplementary Figure 12. Characterization of TRD and non-TRD genes. a Molecular function–GO categories enriched in TRD and non-TRD genes. Adjusted *P*-values were $-\log_{10}$ transformed. **b** Gene-gene distance in TRD and non-TRD genes. Distance of a gene and its closest gene were computed for TRD and non-TRD genes. **c** Distance between a gene and its closest co-regulated enhancer for TRD and non-TRD genes. **d** Distance between a gene and its closest anti-regulated enhancer for TRD and non-TRD genes. **e** TRD and non-TRD genes display similar total mRNA levels (RNA-seq). Boxplot showing distribution of total mRNA levels (Log_2CPM) under the indicated conditions for TRD and non-TRD genes. **f** TRD and non-TRD genes display similar changes of mRNA levels upon TGFβ treatment. Boxplot showing distribution of mRNA levels change (Log_2FC) under the indicated conditions and gene regulatory categories for TRD and non-TRD genes. **e, f** Statistical significance between the indicated distributions were determined with the two-tailed Mann-Whitney non-parametric test. *$p \leq 0.05$. For sample size (n) and exact p-value see Supplementary Data 5. The horizontal black line of the boxplot represents the median value, the box spans the 25th to 75th percentiles, and whiskers indicate 5th and 95th percentiles.

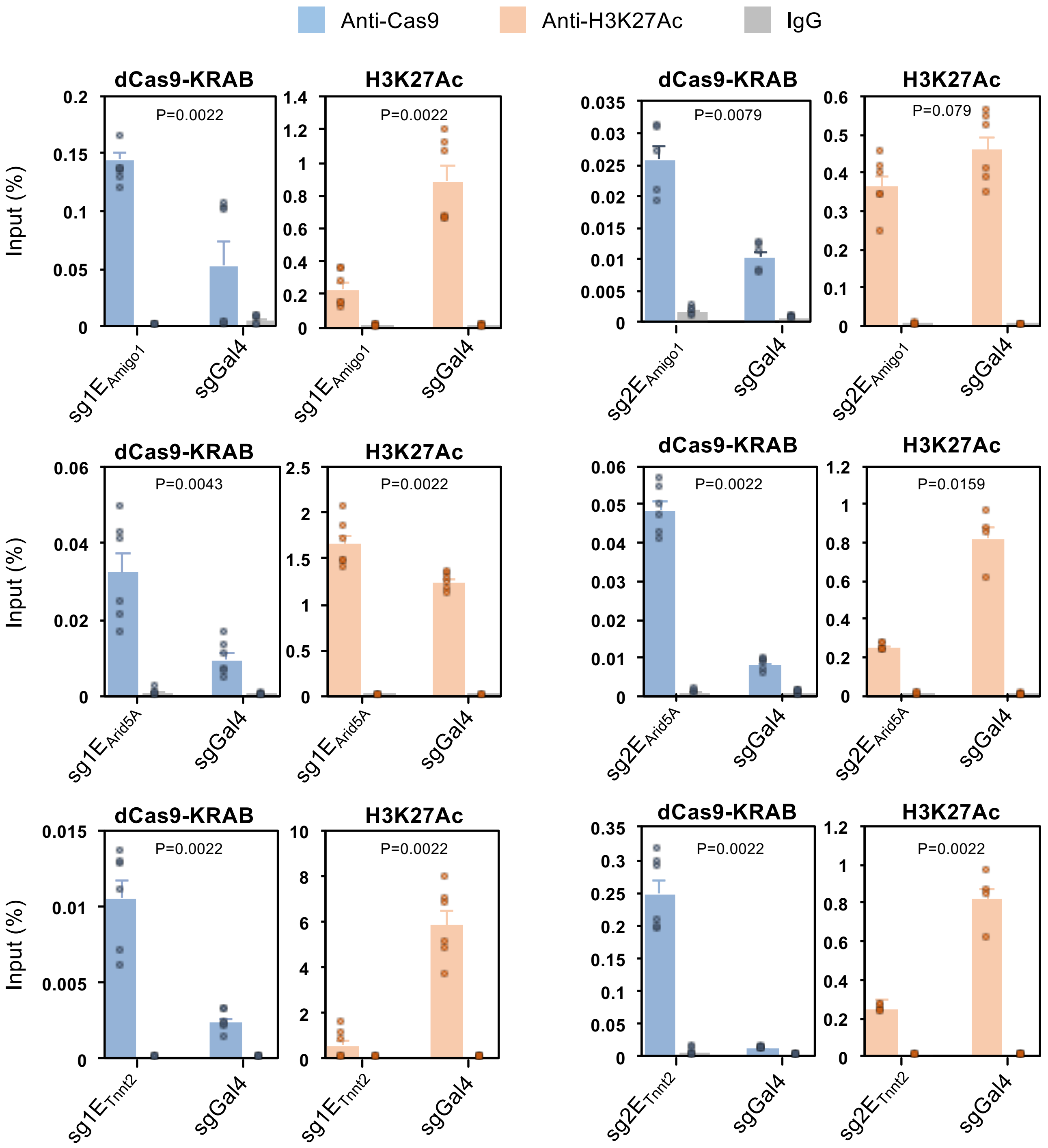

Supplementary Figure 13. Verification by ChIP-PCR of sgRNA targeting and dCas9-KRAB repression activity. ChIP analysis of dCas9-KRAB and H3K27Ac was performed in the NMuMG-derivative cell lines expressing the indicated CRISPR sgRNAs and dCas9-KRAB fusion protein. Map of the investigated enhancers and the regions targeted by the sgRNAs are shown in Figure 6a, c, d. Values are average ± SEM of n ≥ 4 data from three independent experiments. Sample size (n) of each experiments is provided in supplementary Table 5. Statistical significance of the values with respect to the negative control (a non-targeting sgRNA, sgGal4) were determined with the two-tailed Mann-Whitney non-parametric test and p-value are provided.

Supplementary Figure 13

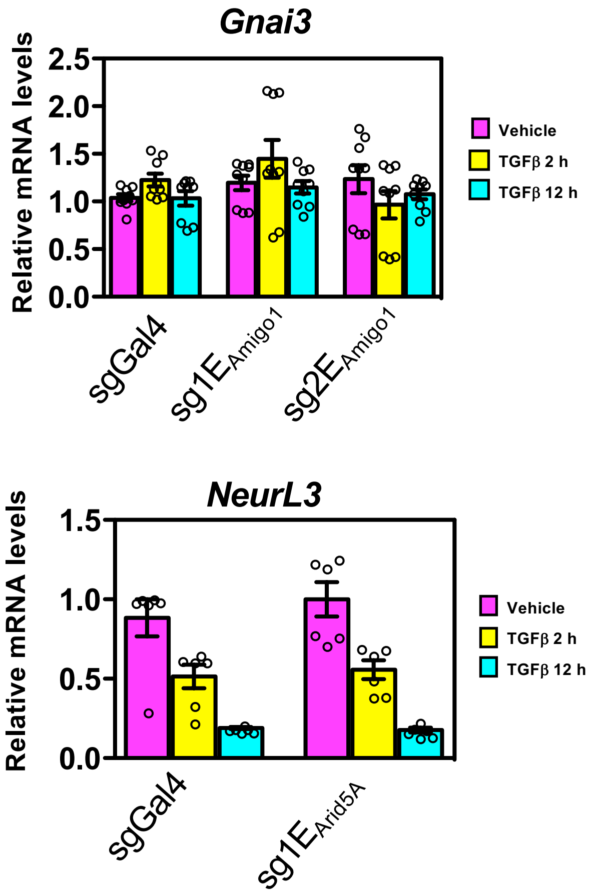

Supplementary Figure 14. **Controls for CRISPR-mediated enhancer inactivation**. Determination of mRNA levels of the indicated genes by RT-qPCR analysis. Values were normalized to *GAPDH* mRNA level. Values are average ± SEM of at least n=6 determinations from three independent experiments. Statistical significance of the values with respect to the same time point of the negative control (a non-targeting sgRNA, sgRNAGal4) were determined by using the two-tailed Mann-Whitney non-parametric test. Differences were not significant. Sample size (n) of each distribution and exact p-values are provided in Supplementary Data 5.

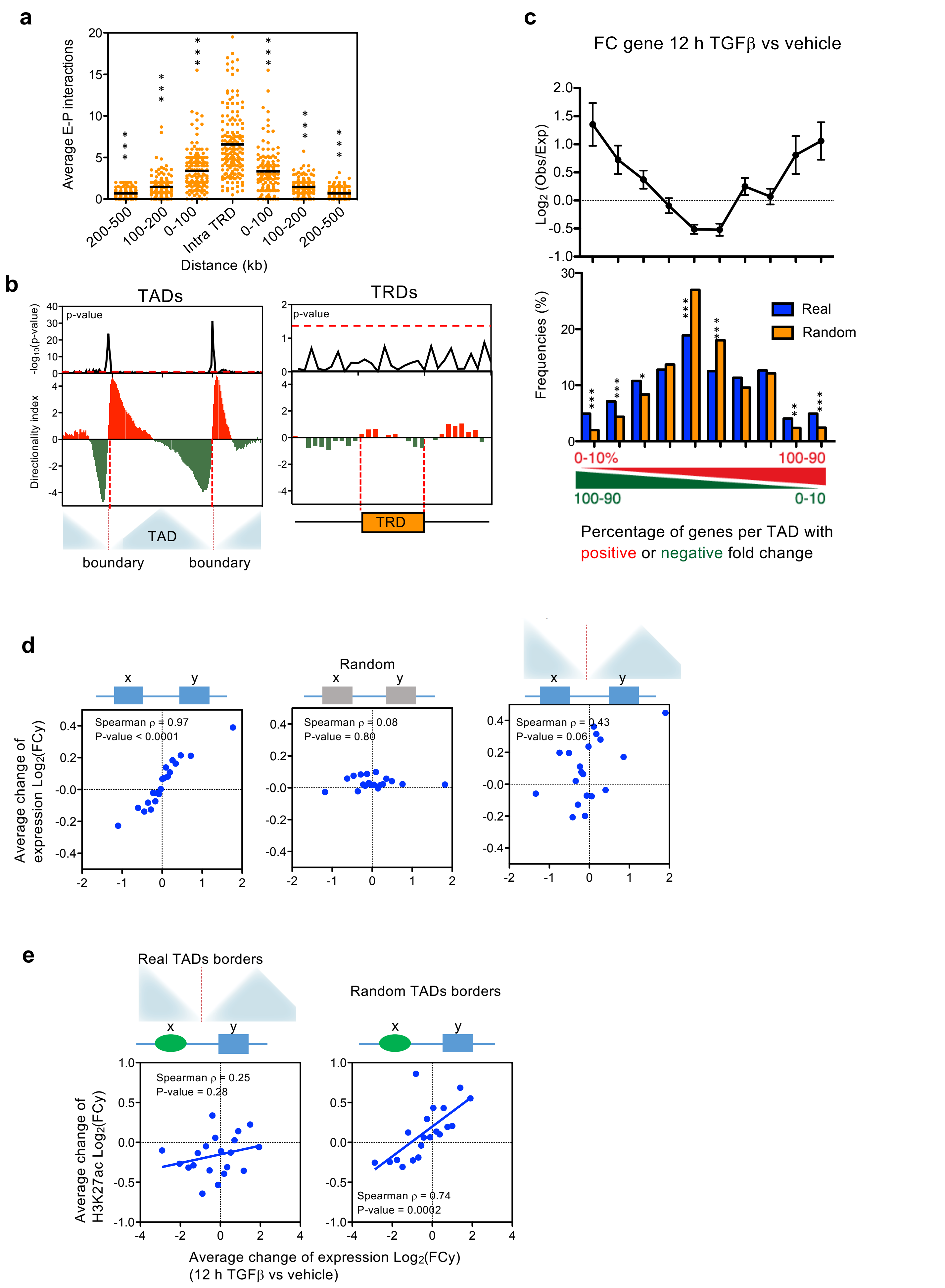

Supplementary Figure 15

Supplementary Figure 15. TGFβ-dependent regulation is affected by TAD borders. **a** Distribution of enhancer-promoter (E-P) Hi-C interactions. The average number of Hi-C interactions between TRD enhancers and promoters of the same TRD (intra-TRD) or promoters placed at the indicated distance from the TRD, were computed. ***$p \leq 0.0001$ in the two-tailed Mann–Whitney Test. Upstream and downstream intervals are compared with the intra-TRD distribution. Sample size (n) and exact p-values are provided in Supplementary Data 5. The horizontal black line of the scatter blot represents the mean value. **b** Meta-TAD and meta-TRD directionality index analysis. Lower panel: Directionality index density was calculated as indicated in Methods. TADs were divided into 100 bins and TRD were divided into 10 bins, proportional to the about 1 Mb and 100 kb average size of each structure. Upper panel: Significance (p-value of two-tailed Mann–Whitney Test) of the difference between two consecutive bins. Red line indicates $p = 0.05$. **a, b** For these analysis Hi-C data were analyzed at 10 kb resolution. **c** Top: ratio of observed versus expected frequencies of TADs with distinct proportions of genes with upregulated or downregulated FC (FC > 1 or FC< 1; TGFβ 12h versus vehicle). Values are means ± SD. Bottom: histogram of TAD frequencies for the observed (blue) or randomized (orange) positions of genes. TADs (n=688) were binned into 10 intervals depending on the percentage of upregulated versus downregulated genes. Significance was determined by comparing the real value with 500 randomizations of the gene order (see Methods). Probabilities of the real number accepting normal distribution are provided. **$p \leq 0.001$; ***$p \leq 0.0001$. For exact p-values see Supplementary Data 5. **d** Correlation between levels of change of expression (TGFβ 2h versus vehicle) between every pair of expressed contiguous genes (x, y) of the genome using real chromosomal order (left), random gene order (middle), or pairs of contiguous genes separated by a TAD border (right). Spearman correlation coefficient and p-value are shown. Data were binned into 20 intervals. **e** Correlation plot between change of H3K27ac signal of enhancers (ChIP-seq signal, TGFβ 12h versus vehicle) and change of mRNA level (RNA-seq signal, TGFβ 12h versus vehicle) of their closest gene separated by a TAD border (left) or using random TAD borders (right). Data were binned into 20 intervals.

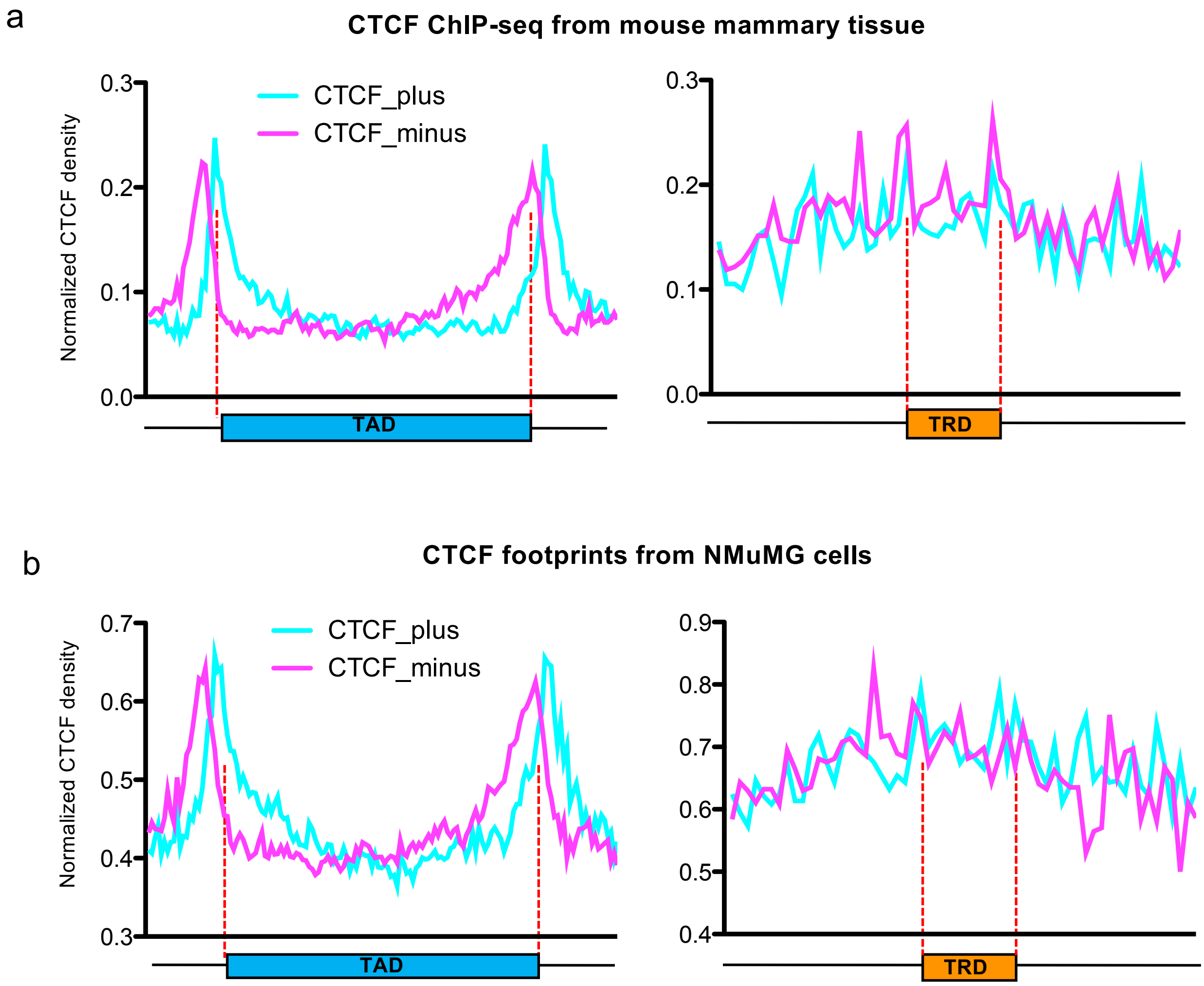

Supplementary Figure 16. CTCF distribution at TAD and TRD borders. a, b Meta-TAD and meta-TRD CTCF density analysis. Density of CTCF binding sites in the plus and in the minus strand is represented. **a** CTCF occupancy was determined by using ChIP-seq data from mouse mammary gland tissue (GEO ID: GSE74826). CTCF motif orientation was determined through CTCF sites identification at the CTCF peaks, by using HOMER. **b** CTCF occupancy and motif orientation was determined using our ATAC-seq footprinting analysis. ATAC-seq reads from the different experimental conditions were pooled in order to have better resolution (similar results were obtained when vehicle, 2h TGFβ and 12h TGFβ data were analyzed separately).

Uncropped versions of western blotting gels

Supplementary Figure 2a

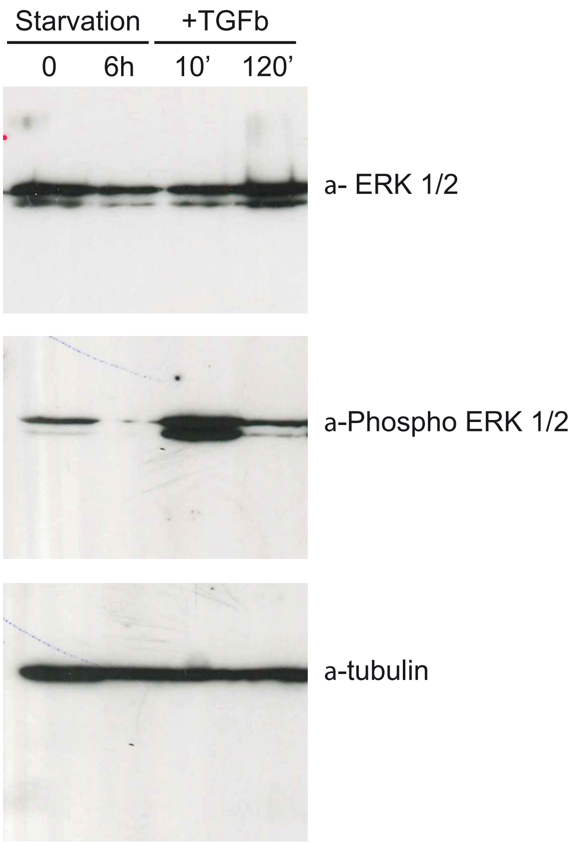

Figure 2g

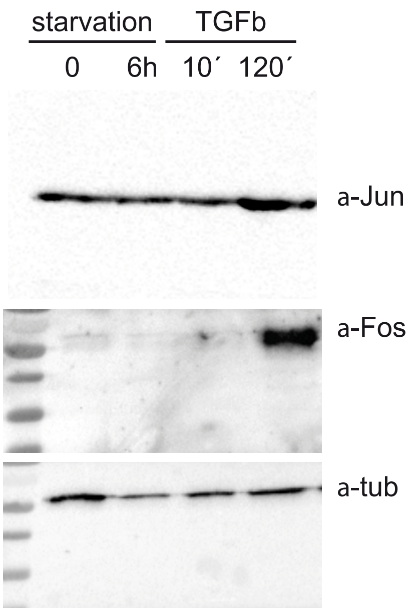

Supplementary Figure 2h

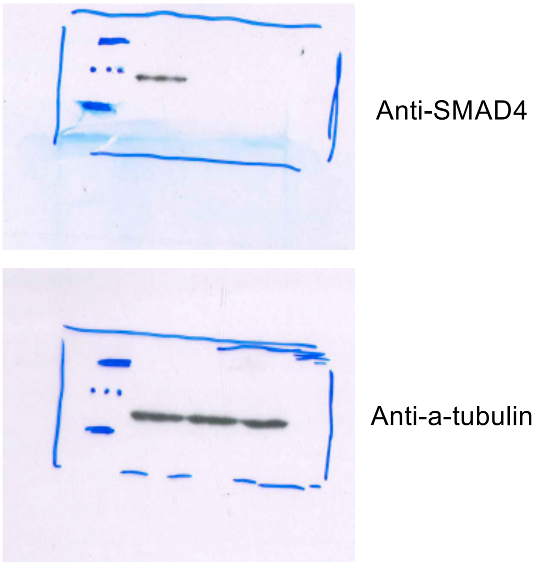

Supplement: Supplementary file 1 — Supplementary Information [file 41467_2020_19877_MOESM1_ESM.pdf]
