# Supplementary material for: TGFβ promotes widespread enhancer chromatin opening and operates on genomic regulatory domains
Source: Nat Commun. 2020 Dec 3;11:6196. doi: 10.1038/s41467-020-19877-5 (PMC7713251; doi:10.1038/s41467-020-19877-5)
Supplement: Supplementary file 3 — Description of Additional Supplementary Files [file 41467_2020_19877_MOESM3_ESM.pdf]

Description of Additional Supplementary Files

Supplementary Data 1. Transcriptomic analysis (RNA-seq). Change of mRNA levels (Log2) and adjusted P-values (using empirical BAYES moderated t-statistics test) of the different comparisons are provided. Sheet 1, all expressed genes; Sheet 2, early upregulated genes; Sheet 3, late upregulated genes; Sheet 4, early downregulated genes; Sheet 5, late downregulated genes; Sheet 6, transient upregulated genes; Sheet 7, transient downregulated genes.

Supplementary Data 2. **Classification and regulation by TGFβ of the enhancers from NMuMG cells.** Chromosome, Coordinates, ATAC peak name, strand, enhancer class previous to TGFβ treatment and enhancer regulation after TGFβ, are provided.

Supplementary Data 3. List of TRDs. Chromosome, TRD coordinates and gene composition are provided. Sheet 1, early upregulated TRDs; Sheet 2, late upregulated TRDs; Sheet 3, early downregulated TRDs; Sheet4, late downregulated TRDs.

Supplementary Data 4. Oligonucleotides and sgRNA sequences. Sheet 1, sgRNAs; Sheet 2, Oligonucleotides for RT-qPCR; Sheet 3, Oligonucleotides for ChIP-PCR.

Supplementary Data 5. Sample size (n) of the distributions and exact p-values from statistical tests.
