# Supplementary material for: TGFβ promotes widespread enhancer chromatin opening and operates on genomic regulatory domains
Source: Nat Commun. 2020 Dec 3;11:6196. doi: 10.1038/s41467-020-19877-5 (PMC7713251; doi:10.1038/s41467-020-19877-5)
Supplement: Supplementary file 9 — Reporting summary [file 41467_2020_19877_MOESM9_ESM.pdf]

natureresearch

DBPR: NCOMMS-19-3156884
Corresponding author(s): Jose C. Reyes

Last updated by author(s): Sep 18, 2020

Reporting Summary

Nature Research wishes to improve the reproducibility of the work that we publish. This form provides structure for consistency and transparency in reporting. For further information on Nature Research policies, seeAuthors & Referees and theEditorial Policy Checklist .

Statistics

For all statistical analyses, confirm that the following items are present in the figure legend, table legend, main text, or Methods section.

n/a	Confirmed	
☐	☒	The exact sample size (*n*) for each experimental group/condition, given as a discrete number and unit of measurement
☐	☒	A statement on whether measurements were taken from distinct samples or whether the same sample was measured repeatedly
☐	☒	The statistical test(s) used AND whether they are one- or two-sided<br>*Only common tests should be described solely by name; describe more complex techniques in the Methods section.*
☐	☒	A description of all covariates tested
☐	☒	A description of any assumptions or corrections, such as tests of normality and adjustment for multiple comparisons
☐	☒	A full description of the statistical parameters including central tendency (e.g. means) or other basic estimates (e.g. regression coefficient) AND variation (e.g. standard deviation) or associated estimates of uncertainty (e.g. confidence intervals)
☐	☒	For null hypothesis testing, the test statistic (e.g. *F*, *t*, *r*) with confidence intervals, effect sizes, degrees of freedom and *P* value noted<br>*Give P values as exact values whenever suitable.*
☒	☐	For Bayesian analysis, information on the choice of priors and Markov chain Monte Carlo settings
☒	☐	For hierarchical and complex designs, identification of the appropriate level for tests and full reporting of outcomes
☒	☐	Estimates of effect sizes (e.g. Cohen's *d*, Pearson's *r*), indicating how they were calculated

Our web collection on statistics for biologists contains articles on many of the points above.

Software and code

Policy information about availability of computer code

Data collection

No software was used for data collection

Data analysis

Software version source
R v3.4.4 https://cran.r-project.org/bin/linux/ubuntu/
RStudio v0.99.879 https://rstudio.com/products/rstudio/download/
Bioconductor v2.38 https://www.bioconductor.org/install/
FastQ toolkit v1.0.0 http://hannonlab.cshl.edu/fastx_toolkit/
encode-ATAC-seq-pipeline https://github.com/ENCODE-DCC/atac-seq-pipeline
csaw v1.12.0 https://bioconductor.org/packages/release/bioc/html/csaw.html
macs2 v2.1.2 https://github.com/taoliu/MACS
bedtools v2.27.1 https://bedtools.readthedocs.io/en/latest/content/installation.html
deeptools v3.1.3 https://deeptools.readthedocs.io/en/develop/content/installation.html
edgeR v3.20.9 https://bioconductor.org/packages/release/bioc/html/edgeR.html
imageJ/Fiji version=1.48k https://imagej.net/Fiji
TANGO v.0.84 https://biophysique.mnhn.fr/tango/HomePage
CentriMo v5.0.2 http://meme-suite.org/doc/download.html
HOMER v4.10.3 http://homer.ucsd.edu/homer/introduction/configure.html
pyDNAse v0.2.4 https://pythonhosted.org/pyDNase/
FIMO v5.0.0 http://meme-suite.org/doc/download.html
Gitools v2.3.1 http://www.gitools.org/download.php
GenomicAlignments v1.20.1 https://bioconductor.org/packages/release/bioc/html/GenomicAlignments.html
Biostrings v2.52.0 https://bioconductor.org/packages/release/bioc/html/Biostrings.html
soGGi v1.16.0 https://www.bioconductor.org/packages/release/bioc/html/soGGi.html

RSubread v1.28.1 https://bioconductor.org/packages/release/bioc/html/Rsubread.html
Voom/limma v3.34.9 https://bioconductor.org/packages/release/bioc/html/limma.html
EnrichedHeatmap v1.12.0 https://bioconductor.org/packages/release/bioc/html/EnrichedHeatmap.html
Shushi v1.16.0 https://www.bioconductor.org/packages/release/bioc/html/Sushi.html
Samtools v0.1.19 http://samtools.sourceforge.net/
HiC-Pro v.2.11.1 https://github.com/nservant/HiC-Pro
PIQ N/A https://github.com/orzechoj/piq-single
vecsets v1.2.1 https://cran.r-project.org/web/packages/vecsets/index.html
stats R package v.3.4.4 https://cran.r-project.org/web/packages/STAT/index.html
UCSC liftover tool N/A https://genome.ucsc.edu/cgi-bin/hgLiftOver

For manuscripts utilizing custom algorithms or software that are central to the research but not yet described in published literature, software must be made available to editors/reviewers. We strongly encourage code deposition in a community repository (e.g. GitHub). See the Nature Research guidelines for submitting code & software for further information.

Data

Policy information about availability of data

All manuscripts must include a data availability statement. This statement should provide the following information, where applicable:

- Accession codes, unique identifiers, or web links for publicly available datasets
- A list of figures that have associated raw data
- A description of any restrictions on data availability

Sequencing data (raw data and processed files) are available at NCBI GEO under the accession code GSE140552.
SMAD2/3 ChIP-seq data was downloaded as a raw data from: GSE121254.
CTCF ChIP-seq data was downloaded as a raw data from GSE74826 .
HiC data was downloaded as a raw data from GSE96033.
Other data will be made available through a Source data file, includding processed data used to construct the figure plots and non-sequencing source data.

Field-specific reporting

Please select the one below that is the best fit for your research. If you are not sure, read the appropriate sections before making your selection.

☒ Life sciences ☐ Behavioural & social sciences ☐ Ecological, evolutionary & environmental sciences

For a reference copy of the document with all sections, see nature.com/documents/nr-reporting-summary-flat.pdf

Life sciences study design

All studies must disclose on these points even when the disclosure is negative.

Sample size	Samples size for each experiment is indicated in the figure legends. The sample size was chosen based on previous experience in the lab, for each experiment to yield high statistics power. For ChIP-seq, ATAC-seq and RNAse-seq n=2 biological replicates were normally performed. ENCODE requires 2 biological replicates for their ChIP-seq analysis (see for example https://www.nature.com/articles/s41586-020-2023-4).<br>Our sample sizes are similar to those generally employed in other relevant similar studies:<br>Guo, H., Ci, X., Ahmed, M. et al, 2019. https://doi.org/10.1038/s41467-018-08133-6<br>Fox, S., Myers, J.A. et al., 2020. https://pubmed.ncbi.nlm.nih.gov/32917861/<br>Li, J., Huang, K., Hu, G. et al, 2019. https://doi.org/10.1038/s41467-019-08949-w<br>No statistical methods were used to predetermine sample size.
Data exclusions	No data were excluded from the analyses
Replication	RT-qPCR experiments were performed in at least 3 biologically independent replicates using two or three technical replicates. Fluorescence microscopy analysis were also performed in at least 3 biologically independent replicates. ChIP-qPCR were performed in at least 2 biologically independent replicates with three technical replicates each one. Western blot experiments were performed in at least 3 biological independent replicates. All replicates were reported in the manuscript. No technical replicates (except for qPCR experiments) were used to calculate statistics. For ChIP-seq, ATAC-seq and RNAse-seq n=2 biological replicates were normally performed. All attempts at replication of the results were successful.
Randomization	This study does not involve randomization of samples, following standard procedures generally employed in other relevant similar studies. See for example Partridge et al., 2020 (https://www.nature.com/articles/s41586-020-2023-4) or Douillet et al., 2020 (https://www.nature.com/articles/s41588-020-0618-1)
Blinding	Investigators were not blinded for selection of cell culture plates used in the treatments (vehicle-treated or TGFbeta-treated) following standard procedures generally employed in other relevant similar studies. See for example Partridge et al., 2020 (https://www.nature.com/articles/s41586-020-2023-4) or Douillet et al., 2020 (https://www.nature.com/articles/s41588-020-0618-1). For image analysis, cell and EM images were quantified in a blinded manner.

Reporting for specific materials, systems and methods

We require information from authors about some types of materials, experimental systems and methods used in many studies. Here, indicate whether each material, system or method listed is relevant to your study. If you are not sure if a list item applies to your research, read the appropriate section before selecting a response.

Materials & experimental systems

n/a	Involved in the study
☐	☒ Antibodies
☐	☒ Eukaryotic cell lines
☒	☐ Palaeontology
☒	☐ Animals and other organisms
☒	☐ Human research participants
☒	☐ Clinical data

Methods

n/a	Involved in the study
☐	☒ ChIP-seq
☒	☐ Flow cytometry
☒	☐ MRI-based neuroimaging

Antibodies

Antibodies used

anti-H3K27ac (Abcam, Ab4729, Lot:GR243994-1)
anti-H3K4me1 (Abcam, Ab8895, Lot: GR243233)
anti-H3K4me3 (Abcam, Ab8580, Lot: GR3201218-1)
anti-alfaTubulin (Sigma-Aldrich, T9026, Lot: 083M4847V)
anti-SMAD4 (Santa Cruz, SC-7996, Lot: G0616)
anti-Erk1/2 (Millipore, 06-182, Lot: 18697)
anti-phospho-Erk1/2 (Cell Signaling, 9101, Rev:09/2012, Lot: 27)
anti-cJun (Cell Signaling, 60A8, Rev:12/2019, Lot: 11)
anti-c-Fos (Cell Signaling, 2250, Rev:11/2019, Lot: 10)
anti-CRISPR-Cas9 (Diagenode, C15310258-100, Lot: A2508-001)
anti-Rabbit IgG (Sigma-Aldrich, I8140, N/A)
anti-mouse-HRP (Sigma-Aldrich, A8786, Lot: 038K4832)
anti-rabbit HRP (Sigma-Aldrich, A0545, Lot: 041K4848)

Validation

anti-H3K27ac:
Xie JJ et al. Super-Enhancer-Driven Long Non-Coding RNA LINC01503, Regulated by TP63, Is Over-Expressed and Oncogenic in Squamous Cell Carcinoma. Gastroenterology 154:2137-2151.e1 (2018)
Mahe M et al. An FGFR3/MYC positive feedback loop provides new opportunities for targeted therapies in bladder cancers. EMBO Mol Med 10:N/A (2018)

anti-H3K4me1:
Xi Y et al. Histone modification profiling in breast cancer cell lines highlights commonalities and differences among subtypes. BMC Genomics 19:150 (2018)
He X et al. A histone deacetylase 3-dependent pathway delimits peripheral myelin growth and functional regeneration. Nat Med 24:338-351 (2018)

anti-H3K4me3:
Zhong J et al. Enhanced and controlled chromatin extraction from FFPE tissues and the application to ChIP-seq. BMC Genomics 20:249 (2019)
Mahe M et al. An FGFR3/MYC positive feedback loop provides new opportunities for targeted therapies in bladder cancers. EMBO Mol Med 10:N/A (2018)

anti-alfaTubulin:
Maschalidi, S., Nunes-Hasler, P., Nascimento, C.R. et al. UNC93B1 interacts with the calcium sensor STIM1 for efficient antigen cross-presentation in dendritic cells. Nat Commun 8, 1640 (2017)

anti-SMAD4:
Rodríguez-Mateo, Cristina et al. “Downregulation of Lnc-Spry1 mediates TGF-β-induced epithelial-mesenchymal transition by transcriptional and posttranscriptional regulatory mechanisms.” Cell death and differentiation vol. 24,5 (2017)

anti-Erk1/2
Frank R, Komiyama N, Ryan T, Zhu F, O Dell T, Grant S. NMDA receptors are selectively partitioned into complexes and supercomplexes during synapse maturation. Nat Commun. 2016;7:11264

anti-phospho-Erk1/2

Ku, A.A., Hu, H., Zhao, X. et al. Integration of multiple biological contexts reveals principles of synthetic lethality that affect reproducibility. Nat Commun 11, 2375 (2020). https://doi.org/10.1038/s41467-020-16078-y
Steins A, van Mackelenbergh MG, van der Zalm AP, et al. High-grade mesenchymal pancreatic ductal adenocarcinoma drives stromal deactivation through CSF-1. EMBO Rep. 2020;21(5):e48780. doi:10.15252/embr.201948780

anti-c-Jun
Li Y, Yu S, Li L, et al. KLF4-mediated upregulation of CD9 and CD81 suppresses hepatocellular carcinoma development via JNK signaling. Cell Death Dis. 2020;11(4):299. Published 2020 Apr 29. doi:10.1038/s41419-020-2479-z

anti-c-Fos
Dohmen M, Krieg S, Agalaridis G, et al. AMPK-dependent activation of the Cyclin Y/CDK16 complex controls autophagy. Nat Commun. 2020;11(1):1032. Published 2020 Feb 25. doi:10.1038/s41467-020-14812-0

anti-CRISPR-Cas9
Zhang Y, Zhang X, Ba Z, et al. The fundamental role of chromatin
Matharu N, Rattanasopha S, Tamura S, et al. CRISPR-mediated activation of a promoter or enhancer rescues obesity caused by haploinsufficiency. Science. 2019;363(6424):eaau0629. doi:10.1126/science.aau0629 loop extrusion in physiological V(D)J recombination. Nature. 2019;573(7775):600-604. doi:10.1038/s41586-019-1547-y

Eukaryotic cell lines

Policy information about cell lines

Cell line source(s)

NMuMG cell line was provided by Jose Antonio Pintor-Toro (commercial source: ATCC)
MCF7 cell lines was provided by Abelardo Lopez-Rivas (commercial source: ATCC)
RPE1 cell line was provided by Rosa María Rios-Sánchez (commercial source: ATCC)
HEK293T cell line was provided by Rosa María Rios-Sánchez (commercial source: ATCC)

Authentication

NMuMG was validated as a mouse cell line through our Next Generation Sequencing experiments. Moreover, both the epithelial phenotype and the kinetic of the induction to the mesenchymal phenotype with TGFbeta were assessed in our experiments many times through immunofluorescence, western blotting and RT-qPCR analysis of epithelial to mesenchymal transition markers assays (SNAI1, VIM, FN1, CDH1). All these characteristics are typical and some of them unique of NMuMG cells. MCF7, RPE-1 and HEK293T cell lines were no authenticated.

Mycoplasma contamination

All cell lines were tested negative for mycoplasma contamination

Commonly misidentified lines
(See ICLAC register)

No commonly misidentified cell lines was used in this study

ChIP-seq

Data deposition

- [x] Confirm that both raw and final processed data have been deposited in a public database such as GEO.
- [x] Confirm that you have deposited or provided access to graph files (e.g. BED files) for the called peaks.

Data access links
May remain private before publication.

GEO accesion number: GSE140552

Files in database submission

./DNaseI-seq/processed_data/DNAse_TGFb2h_peaks.bed
./DNaseI-seq/processed_data/DNAse_veh_peaks.bed
./DNaseI-seq/raw_data/ DNAse_TGFb2h_merged_R1.fastq.gz
./DNaseI-seq/raw_data/DNAse_TGFb2h_merged_R2.fastq.gz
./DNaseI-seq/raw_data/DNAse_veh_merged_R1.fastq.gz
./DNaseI-seq/raw_data/DNAse_veh_merged_R2.fastq.gz
./ATAC-seq/processed_data/ATAC_TGFb10m_peaks.bed
./ATAC-seq/processed_data/ATAC_TGFb2h_FBS.bed
./ATAC-seq/processed_data/ATAC_veh_FBS_peaks.bed
./ATAC-seq/raw_data/ATAC_TGFb10m_exp10m_R1.fastq.gz
./ATAC-seq/raw_data/ATAC_TGFb10m_exp10m_R2.fastq.gz
./ATAC-seq/raw_data/ATAC_TGFb2h_FBS_R1.fastq.gz
./ATAC-seq/raw_data/ATAC_TGFb2h_FBS_R2.fastq.gz
./ATAC-seq/raw_data/ATAC_veh_exp10m_R1.fastq.gz
./ATAC-seq/raw_data/ATAC_veh_exp10m_R2.fastq.gz
./ATAC-seq/raw_data/ATAC_veh_FBS_R1.fastq.gz
./ATAC-seq/raw_data/ATAC_veh_FBS_R2.fastq.gz
./ATAC-seq/processed_data/ATAC_TGFb12h.bed
./ATAC-seq/processed_data/ATAC_TGFb2h.bed
./ATAC-seq/processed_data/ATAC_veh.bed

./ATAC-seq/raw_data/TGFb12hr1_1.fastq.gz
./ATAC-seq/raw_data/TGFb12hr1_2.fastq.gz
./ATAC-seq/raw_data/TGFb12hr2_1.fastq.gz
./ATAC-seq/raw_data/TGFb12hr2_2.fastq.gz
./ATAC-seq/raw_data/TGFb2hr1_1.fastq.gz
./ATAC-seq/raw_data/TGFb2hr1_2.fastq.gz
./ATAC-seq/raw_data/TGFb2hr2_1.fastq.gz
./ATAC-seq/raw_data/TGFb2hr2_2.fastq.gz
./ATAC-seq/raw_data/vehr1_1.fastq.gz
./ATAC-seq/raw_data/vehr1_2.fastq.gz
./ATAC-seq/raw_data/vehr2_1.fastq.gz
./ATAC-seq/raw_data/vehr2_2.fastq.gz
./ChIP-seq/processed_data/input_TGFb12h_r1.bw
./ChIP-seq/processed_data/input_TGFb12h_r2.bw
./ChIP-seq/processed_data/input_TGFb2h_r1.bw
./ChIP-seq/processed_data/input_TGFb2h_r2.bw
./ChIP-seq/processed_data/input_veh_r1.bw
./ChIP-seq/processed_data/input_veh_r2.bw
./ChIP-seq/processed_data/K27ac_TGFb12h_r1.bw
./ChIP-seq/processed_data/K27ac_TGFb12h_r2.bw
./ChIP-seq/processed_data/K27ac_TGFb2h_r1.bw
./ChIP-seq/processed_data/K27ac_TGFb2h_r2.bw
./ChIP-seq/processed_data/K27ac_veh_r1.bw
./ChIP-seq/processed_data/K27ac_veh_r2.bw
./ChIP-seq/processed_data/K4me1_TGFb12h_r1.bw
./ChIP-seq/processed_data/K4me1_TGFb12h_r2.bw
./ChIP-seq/processed_data/K4me1_TGFb2h_r1.bw
./ChIP-seq/processed_data/K4me1_TGFb2h_r2.bw
./ChIP-seq/processed_data/K4me1_veh_r1.bw
./ChIP-seq/processed_data/K4me1_veh_r2.bw
./ChIP-seq/processed_data/K4me3_TGFb12h_r1.bw
./ChIP-seq/processed_data/K4me3_TGFb12h_r2.bw
./ChIP-seq/processed_data/K4me3_TGFb2h_r1.bw
./ChIP-seq/processed_data/K4me3_TGFb2h_r2.bw
./ChIP-seq/processed_data/K4me3_veh_r1.bw
./ChIP-seq/processed_data/K4me3_veh_r2.bw
./ChIP-seq/raw_data/input_TGFb12h_r1.fastq.gz
./ChIP-seq/raw_data/input_TGFb12h_r2.fastq.gz
./ChIP-seq/raw_data/input_TGFb2h_r1.fastq.gz
./ChIP-seq/raw_data/input_TGFb2h_r2.fastq.gz
./ChIP-seq/raw_data/input_veh_r1.fastq.gz
./ChIP-seq/raw_data/input_veh_r2.fastq.gz
./ChIP-seq/raw_data/K27ac_TGFb12h_r1.fastq.gz
./ChIP-seq/raw_data/K27ac_TGFb12h_r2.fastq.gz
./ChIP-seq/raw_data/K27ac_TGFb2h_r1.fastq.gz
./ChIP-seq/raw_data/K27ac_TGFb2h_r2.fastq.gz
./ChIP-seq/raw_data/K27ac_veh_r1.fastq.gz
./ChIP-seq/raw_data/K27ac_veh_r2.fastq.gz
./ChIP-seq/raw_data/K4me1_TGFb12h_r1.fastq.gz
./ChIP-seq/raw_data/K4me1_TGFb12h_r2.fastq.gz
./ChIP-seq/raw_data/K4me1_TGFb2h_r1.fastq.gz
./ChIP-seq/raw_data/K4me1_TGFb2h_r2.fastq.gz
./ChIP-seq/raw_data/K4me1_veh_r1.fastq.gz
./ChIP-seq/raw_data/K4me1_veh_r2.fastq.gz
./ChIP-seq/raw_data/K4me3_TGFb12h_r1.fastq.gz
./ChIP-seq/raw_data/K4me3_TGFb12h_r2.fastq.gz
./ChIP-seq/raw_data/K4me3_TGFb2h_r1.fastq.gz
./ChIP-seq/raw_data/K4me3_TGFb2h_r2.fastq.gz
./ChIP-seq/raw_data/K4me3_veh_r1.fastq.gz
./ChIP-seq/raw_data/K4me3_veh_r2.fastq.gz
./ChromRNA-seq/processed_data/Ch-RNA_TGFb12h_r1.bw
./ChromRNA-seq/processed_data/Ch-RNA_TGFb12h_r2.bw
./ChromRNA-seq/processed_data/Ch-RNA_TGFb2h_r1.bw
./ChromRNA-seq/processed_data/Ch-RNA_TGFb2h_r2.bw
./ChromRNA-seq/processed_data/Ch-RNA_veh_r1.bw
./ChromRNA-seq/processed_data/Ch-RNA_veh_r2.bw
./ChromRNA-seq/raw_data/ChromRNA_TGFb12h_r1.fastq.gz
./ChromRNA-seq/raw_data/ChromRNA_TGFb12h_r2.fastq.gz

./ChromRNA-seq/raw_data/ChromRNA_TGFb2h_r1.fastq.gz
./ChromRNA-seq/raw_data/ChromRNA_TGFb2h_r2.fastq.gz
./ChromRNA-seq/raw_data/ChromRNA_veh_r1.fastq.gz
./ChromRNA-seq/raw_data/ChromRNA_veh_r2.fastq.gz
./RNA-seq/processed_data/raw_counts.txt
./RNA-seq/raw_data/RNA_TGFb12h_r1.fastq.gz
./RNA-seq/raw_data/RNA_TGFb12h_r2.fastq.gz
./RNA-seq/raw_data/RNA_TGFb2h_r1.fastq.gz
./RNA-seq/raw_data/RNA_TGFb2h_r2.fastq.gz
./RNA-seq/raw_data/RNA_veh_r1.fastq.gz
./RNA-seq/raw_data/RNA_veh_r2.fastq.gz

Genome browser session
(e.g. UCSC)

http://genome.ucsc.edu/s/Alicia%20Subtil%2DRodriguez/NatComms2

Methodology

Replicates

To perform ChIP-seq experiments two independent biological replicates was used.

Sequencing depth

ChIP-seq experiments are single-end with a read size of 50 bp.
name Total Uniquely_Mapped
bam/input_veh_r1.bam 74067701 58444326
bam/input_veh_r2.bam 41554381 31069808
bam/input_TGFb2h_r1.bam 72160972 61225409
bam/input_TGFb2h_r2.bam 39655845 33008486
bam/input_TGFb12h_r1.bam 32375614 19469394
bam/input_TGFb12h_r2.bam 33858162 21319561
bam/K4me1_veh_r1.bam 39878573 36024616
bam/K4me1_veh_r2.bam 50695142 36751888
bam/K4me1_TGFb2h_r1.bam 41220386 37574065
bam/K4me1_TGFb2h_r2.bam 56343861 46575076
bam/K4me1_TGFb12h_r1.bam 50166901 35807165
bam/K4me1_TGFb12h_r2.bam 34104120 28991314
bam/K4me3_veh_r1.bam 45726114 37276693
bam/K4me3_veh_r2.bam 69608088 57426887
bam/K4me3_TGFb2h_r1.bam 42046893 34841047
bam/K4me3_TGFb2h_r2.bam 51295337 43225558
bam/K4me3_TGFb12h_r1.bam 52810793 39659636
bam/K4me3_TGFb12h_r2.bam 43741215 33048218
bam/K27ac_veh_r1.bam 102016983 87885852
bam/K27ac_veh_r2.bam 30890168 28260473
bam/K27ac_TGFb2h_r1.bam 88044547 73109659
bam/K27ac_TGFb2h_r2.bam 59772824 55365711
bam/K27ac_TGFb12h_r1.bam 51124775 38443872
bam/K27ac_TGFb12h_r2.bam 55651664 40652148

ATAc-seq and DNaseI-seq experiments are paired-end with a read size of 75 bp.
name Total_paired_reads Mapped_and_filtered_reads
bam/veh_r1.bam 213547366 128768960
bam/veh_r2.bam 174689786 100702656
bam/TGFb2h_r1.bam 208937984 106610414
bam/TGFb2h_r2.bam 186034704 103808154
bam/TGFb12h_r1.bam 216396636 90260358
bam/TGFb12h_r2.bam 171429808 67617626
bam/ATAC_veh_exp10m.bam 54893460 34208424
bam/ATAC_TGFb10m_exp10m.bam 65587840 36325876
bam/ATAC_veh_FBS.bam 78840800 48533492
bam/ATAC_TGFb2h_FBS.bam 75764908 42429056
bam/DNAse_veh.bam 90208426 59660580
bam/DNAse_TGFb2h.bam 57727518 37824966

Antibodies

anti-H3K27ac (Abcam, Ab4729, Lot:GR243994-1)
anti-H3K4me1 (Abcam, Ab8895, Lot: GR243233)
anti-H3K4me3 (Abcam, Ab8580, Lot: GR3201218-1)

Peak calling parameters

No peak calling was performed for ChIP-seq data, instead of ChIP-seq data were measured in the set of ATAC-seq peaks

Data quality

FastQC was used to analyze sequencing quality for raw ChIP-seq data. As no peak calling was performed there is no information about FDR to report. To determine ChIP-seq differential analyses we considered as statistically significant all peaks with a log2(FC) larger than 1 with a p-value smaller than 0.05.

Software

Most of analyses was performed using R (v3.4.4), RStudio (v.099.879) and Bioconductor (v2.38). To ChIP-seq alignment we used align() function from RSubread package (v1.28.1) with type = 1, TH1 = 2 and unique = TRUE parameters. Samtools rmdup was used to remove duplicates. For differential binding analysis csaw (v1.12.0) and edgeR (v3.20.9) packages were used. bamCoverage from deeptools (v3.1.3) was used to convert bam files into bigWig using --normalizeRPKM option.
